# Personality, cognition and behavior in chimpanzees: a new approach based on Eysenck’s model

**DOI:** 10.7717/peerj.9707

**Published:** 2020-08-17

**Authors:** Maria Padrell, David Riba, Yulán Úbeda, Federica Amici, Miquel Llorente

**Affiliations:** 1 Facultat d’Educació i Psicologia, Universitat de Girona, Girona, Spain; 2 Unitat de Recerca i Etologia, Fundació Mona, Girona, Spain; 3 Facultat de Lletres, Universitat de Girona, Girona, Spain; 4 Research Group “Primate Behavioural Ecology”, Department of Human Behavior, Ecology and Culture, Max-Planck Institute for Evolutionary Anthropology, Leipzig, Germany; 5 IPRIM, Institut de Recerca i Estudis en Primatologia, Girona, Spain

**Keywords:** Chimpanzees, Cognition, Cognitive research, Performance, Personality, Behavior

## Abstract

Personality has been linked to individual variation in interest and performance in cognitive tasks. Nevertheless, this relationship is still poorly understood and has rarely been considered in animal cognition research. Here, we investigated the association between personality and interest, motivation and task performance in 13 sanctuary chimpanzees (*Pan troglodytes*) housed at Fundació Mona (Spain). Personality was assessed with a 12-item questionnaire based on Eysenck’s Psychoticism-Extraversion-Neuroticism model completed by familiar keepers and researchers. Additionally, personality ratings were compared to behavioral observations conducted over an 11-year period. Experimental tasks consisted in several puzzle boxes that needed to be manipulated in order to obtain a food reward. Dependent variables included participation (as an indicator of interest), success and latency (as measures of performance), and losing contact with the task (as an indicator of motivation). As predicted, we obtained significant correlations between Eysenck’s personality traits and observed behaviors, although some expected associations were absent. We then analyzed data using Generalized Linear Mixed Models, running a model for each dependent variable. In both sexes, lower Extraversion and lower Dominance were linked to a higher probability of success, but this effect was stronger in females. Furthermore, higher Neuropsychoticism predicted higher probability of success in females, but not in males. The probability of losing contact with the task was higher in young chimpanzees, and in those rated lower on Extraversion and higher on Dominance. Additionally, chimpanzees rated higher on Neuropsychoticism were also more likely to stop interacting with the task, but again this was more evident in females. Participation and latency were not linked to any personality trait. Our findings show that the PEN may be a good model to describe chimpanzee personality, and stress the importance of considering personality when interpreting the results of cognitive research in non-human primates.

## Introduction

Research on animal personality has been defined as behavioral inter-individual differences consistent over time and across contexts (Réale et al., 2007), and is a field of growing interest, both from a theoretical and an applied perspective. To date, there is evidence that personality traits in non-human animals are similar to those describing human personality (Gosling, 2001; Sih et al., 2004), and that these traits share common neurophysiological substrates (Carere, Caramaschi & Fawcett, 2010; Koolhaas et al., 2010; Latzman et al., 2015). From an evolutionary point of view, behavioral variation across individuals can generate differences in terms of fitness, and is therefore subject to natural selection (Réale et al., 2010; Smith & Blumstein, 2008). The study of animal personality can therefore help us to better understand why subjects may respond differently when they face similar conditions (Carere & Maestripieri, 2013), thus becoming an important contribution to the fields of animal behavior and cognition (Carere & Locurto, 2011; Griffin, Guillette & Healy, 2015; Guillette, Naguib & Griffin, 2017). In fact, associations between personality and performance in cognitive contexts have been documented in a wide range of taxa, including fish (Kareklas, Elwood & Holland, 2017; White et al., 2017), birds (Amy, Van Oers & Naguib, 2012; Medina-García, Jawor & Wright, 2017), ungulates (Nawroth, Prentice & McElligott, 2017) and canids (Svartberg, 2002).

Personality has been broadly studied in non-human primates (Freeman & Gosling, 2010; Weiss, King & Murray, 2011), since our closest living relatives constitute an excellent model for comparative research, thus providing insight on the evolutionary origins of human personality (Figueredo et al., 2015; Michalski & Shackelford, 2010). Firstly, most non-human primates exhibit complex social structures and behaviors, which likely favored the emergence of individual differences (Adams et al., 2015; Mitani et al., 2012). Secondly, their phylogenetic closeness to humans allows us to better understand and rate their personality traits using questionnaires (Weiss & Adams, 2013). Since one of the most relevant attempts to describe chimpanzee personality using a human model with a hierarchical structure (King & Figueredo, 1997), several studies in captivity and in the wild have shown that chimpanzees have specific personality dimensions or traits that are common to their species (King, Weiss & Farmer, 2005; Weiss et al., 2012; Weiss et al., 2017), and that questionnaires adapted from human models are reliable measures of their personality (Freeman et al., 2013; Úbeda & Llorente, 2015; Weiss & Adams, 2013; Weiss et al., 2009; Weiss et al., 2017). Moreover, several studies have reported correlations between trait rating and observed behavior, both in monkeys (Ebenau et al., 2019; Iwanicki & Lehmann, 2015) and in great apes (Eckardt et al., 2015; Murray, 2011; Schaefer & Steklis, 2014; Vazire et al., 2007; Pederson, King & Landau, 2005; Konečná et al., 2008), thus confirming that personality ratings can successfully predict individual behavior. Nonetheless, the use of a rating methodology is not without limitations. On the one hand, some authors have identified a bias between personality ratings and behavioral coding (Uher & Asendorpf, 2008; Highfill et al., 2010; Uher & Visalberghi, 2016). On the other hand, most studies finding a correlation between both methods only obtained partial convergent validity, and very limited discriminant validity (see Šlipogor et al., 2020). In other words, not all the expected traits associate with specific behaviors; some traits may correlate with several behaviors or some behaviors with more than one trait (Capitanio, 2004).

The vast majority of studies assessing personality in non-human primates have used the Hominoid Personality Questionnaire or HPQ (Weiss et al., 2009), which is based on the human Five Factor Model (Goldberg, 1990). The HPQ constitutes a complex personality model consisting of 54 adjectives to rate, which describes five personality traits homologous to the human traits in the Five Factor Model (FFM): Neuroticism, Extraversion, Agreeableness, Conscientiousness, and Openness (to Experience). In addition, the HPQ further contains the trait Dominance, which was described for the first time in chimpanzees (King & Figueredo, 1997). More recently, other authors have applied other top-down human models to study chimpanzee personality, such as Eysenck’s Psychoticism-Extraversion-Neuroticism model (Úbeda & Llorente, 2015) or Cattell’s 16 PF (Ortín et al., 2019). The Eysencks’ psychobiological theory (Eysenck & Eysenck, 1964; Eysenck & Eysenck, 1975) is focused on the underlying biological mechanisms of personality dimensions. On this matter, higher-order traits (Psychoticism, Extraversion and Neuroticism; PEN model) are based on genetic (Eaves et al., 1989) and neurobiological factors (e.g., extraverts present low arousal levels at the ascending reticular activation system; Eysenck, 1967; Eysenck, 1997). Both the FFM and the PEN model have been empirically validated and can be easily integrated. In fact, they share two common dimensions or traits (Neuroticism and Extraversion); and the third trait described by Eysenck, Psychoticism, has been negatively related to Agreeableness and Conscientiousness in the FFM (Goldberg & Rosolack, 1994; Zuckerman et al., 1993). According to Eysenck (1991), however, Conscientiousness, Agreeableness and Openness in the FFM were not major components of personality, but rather represented compounds of what he considered the three higher-order traits. Nonetheless, some authors have found moderate correlations between Openness and Eysenck’s Extraversion (Vorkapić, 2012). Additionally, Goldberg & Rosolack (1994) found a link between Goldberg’s clusters and the PEN model. In particular, they showed that in Openness’ clusters such us intellectuality, depth and foresight, the presumed PEN factor is E- (lower Extraversion); while for the clusters intelligence, nonconformity, sophistication or curiosity, the presumed PEN factor is E+ (high Extraversion).

In their assessment of the PEN model to describe chimpanzees’ personality, Úbeda & Llorente (2015) adapted a 12-item questionnaire rated on a 7-point Likert scale. The authors identified three dimensions: Extraversion, Neuropsychoticism and Dominance. The adjectives that loaded onto Extraversion were very similar to those reported for humans in that same dimension, thus facilitating the interpretation of this trait. Conversely, they identified a compound dimension including adjectives that in humans loaded onto both Neuroticism and Psychoticism, and was therefore labeled Neuropsychoticism. Finally, the authors identified a third factor, which was denominated Dominance, because the adjectives that loaded onto this trait were among those reported in previous studies for Dominance in chimpanzees (King & Figueredo, 1997). This dimension is not directly comparable with any human trait, but it has been repeatedly described in chimpanzees (Freeman & Gosling, 2010; King & Figueredo, 1997) and other non-human primates (Adams et al., 2015; Weiss et al., 2011).

In addition to defining personality traits for each species, studies in non-human primates have allowed researchers to evaluate the influence of personality on critical aspects of animals’ life, such as health (Robinson et al., 2018), welfare (Robinson et al., 2017) and longevity (Altschul et al., 2018; Weiss et al., 2013). Moreover, several studies have explored the link between personality and cognitive performance in non-human primates, using a variety of experimental tasks and performance measures. The trait Openness for instance, has been linked to training success in both capuchin monkeys (*Sapajus apella*: Morton, Lee & Buchanan-Smith, 2013) and chimpanzees (*Pan troglodytes*: Reamer et al., 2014). Similarly, Wergård et al. (2016) reported that the personality trait Activity was positively associated with training success in long-tailed macaques (*Macaca fascicularis*). In more cognitively demanding situations, some studies have also reported a positive association between Openness and chimpanzees’ participation and performance in computerized activities (Altschul et al., 2017; Herrelko, Vick & Buchanan-Smith, 2012) and foraging puzzles (Hopper et al., 2014). Furthermore, Altschul, Terrace & Weiss (2016) reported that rhesus macaques (*Macaca mulatta*) scoring higher in Openness and Friendliness performed better in serial learning tasks. Additionally, when presented with foraging puzzles, male chimpanzees rated higher on Dominance spent more time interacting with the puzzles (Hopper et al., 2014). Conversely, Altschul et al. (2017) concluded that Dominance did not have a major impact on chimpanzees’ participation and performance in the computer-based tasks that they tested. Nevertheless, they found that chimpanzees with high Conscientiousness consistently participated more, performed better and were less likely to drop, although this could depend on their preexisting experience with the task (Altschul et al., 2017).

In humans, Conscientiousness has been repeatedly associated with academic achievement (Noftle & Robins, 2007; Von Stumm, Hell & Chamorro-Premuzic, 2011) and job performance (Hurtz & Donovan, 2000; Mount, Barrick & Strauss, 1999; Rrick & Mount, 1991). Conscientious individuals tend to be more goal-oriented and plan more, and they are better able to delay gratification (Roberts et al., 2009). Furthermore, according to several studies, Conscientiousness in the FFM negatively correlates with Psychoticism in the PEN model (Eysenck, 1992), which would explain the negative impact of Psychoticism on academic performance (Flores-Mendoza et al., 2013; Heaven, Ciarrochi & Vialle, 2007; Poropat, 2011). However, Psychoticism has also been consistently linked to creativity (Abraham et al., 2005; Acar & Runco, 2012; Eysenck, 1995). Regarding other personality dimensions present both in the FFM and in the PEN model, like Extraversion or Neuroticism, studies in human and non-human primates are inconsistent, although there are some exceptions worth noting in humans. For example, several authors have demonstrated a link between higher Neuroticism and poorer performance in cognitive tests, either in academic (Chamorro-Premuzic & Furnham, 2003) or non-academic contexts (Dobson, 2000; Reynolds, McClelland & Furnham, 2014). This has been mainly attributed to the fact that highly neurotic individuals are more likely to experience anxiety when exposed to uncertain or stressful situations. To our knowledge, studies in non-human primates have not detected any significant impact of Neuroticism on cognitive performance. However, Hopper et al. (2014) found that while performing cognitive tasks, chimpanzees with higher Neuroticism exhibited more self-directed behaviors, which are a common indicator of anxiety in both catarrhine (Maestripieri et al., 1992) and platyrrhine primates (Manson & Perry, 2000). Finally, in humans, higher Extraversion has been linked to lower academic achievement, presumably because introverts have a focused, goal-oriented attention, and therefore are less easily distracted (Entwistle & Entwistle, 1970), whereas extraverts have selective, stimulus-oriented attention and prefer to focus on social activities (Sánchez, Rejano & Rodríguez, 2001; Fishman, Ng & Bellugi, 2011). It has also been suggested that extraverts and introverts may show different performance depending on the context (Cox-Fuenzalida et al., 2006). That is to say, extraverts naturally possess low levels of cortical arousal and therefore they perform better in stimulating environments, while introverts are characterized by high levels of cortical arousal and tend to be less efficient when facing an exciting stimulus, but are more successful at task of longer duration (Eysenck, 1983; Eysenck & Eysenck, 1985; Li et al., 2010).

In view of the scant literature exploring the relationship between personality and performance in non-human primates, and of the controversial results reported so far, the main aims of this study were to (i) assess the correspondence between the personality traits from the PEN model (previously adapted by Úbeda & Llorente (2015)) and chimpanzees’ spontaneous behavior, and (ii) evaluate whether individual differences in chimpanzees’ personality are linked to their interest, motivation and performance in cognitive tasks. Firstly, we expected to find significant correlations between personality traits and behaviors that match the definitions of the traits (e.g., Extraversion positively correlating with social behaviors and with affiliative interactions, such as grooming and social play; Dominance with agonistic dominance; and Neuropsychoticism with agonistic behaviors and with behaviors related to anxiety, such as self-directed behaviors or abnormal behaviors). Secondly, considering that Eysenck’s personality traits have been previously linked to cognitive performance in humans, we expected to detect similar associations in chimpanzees. In particular, we predicted that, chimpanzees with higher scores on Extraversion would be more interested in participating in the experimental sessions, as we would expect extraverted individuals to be more curious towards a novel stimulus. However, introverts’ focused attention and lower distractibility are highly desirable attributes to be successful in complex tasks such as the ones presented in this study. Therefore we predicted that higher Extraversion would be related to lower success. Additionally, we expected that chimpanzees rated higher on Neuropsychoticism would also be less successful at solving the tasks, as well as more likely to lose motivation, because they would be less patient and more prone to feel anxious during the experimental sessions. Finally, we predicted that Dominance would not play a determinant role in chimpanzees’ performance, as previously shown by Altschul et al. (2017) and Hopper et al. (2014) when assessing complex cognitive tasks in this species. Moreover, considering that previous studies have shown distinct associations between personality traits and performance in male and female chimpanzees (Hopper et al., 2014), we also decided to explore sex differences.

## Materials & Methods

### Subjects and study site

The study sample consisted of 14 chimpanzees (*Pan troglodytes*), 9 males and 5 females, that ranged in age from 6 to 27 years (mean age  ± SD = 17.71  ± 7.46 years) at the beginning of the study period. They were housed at Fundació Mona (Girona, Spain), a center dedicated to the rescue, rehabilitation and re-socialization of primates that have been previously used as pets or for entertainment. The chimpanzees lived in two separate groups, which have been mostly stable over the years. Under good weather conditions, the chimpanzees spend daytime hours in a 5,640 m^2^ outdoor enclosure, divided into two areas (2,420 m^2^ and 3,220 m^2^), one for each group. The enclosure is covered by natural grasses and other Mediterranean herbaceous vegetation subject to seasonal changes, and contains artificial elements such as wooden platforms, towers and ropes. Besides the exterior enclosures, the chimpanzees also have access to 140 m^2^ indoor facilities in which they spend the nights and rainy/cold days. Additionally, there are two 25 m^2^ exterior cages containing physical enrichment elements, such as ropes and hammocks, which are used to host newly arrived individuals before their integration in a social group. As explained below, the chimpanzees were isolated in this area during the experimental sessions.

### Personality ratings

Personality was assessed using a questionnaire based on the Psychoticism-Extraversion-Neuroticism (PEN) model of personality (Eysenck, 1967). This tool was used for the first time in chimpanzees in a previous study at Fundació Mona (Úbeda & Llorente, 2015). Therefore, 10 of the 14 individuals of our sample had been previously assessed in 2012 using this questionnaire. As described in Úbeda & Llorente (2015), the PEN questionnaire consisted of 12 adjectives rated on a 7-point Likert scale. A brief definition for each trait was also included at the end of the document. An English translation of the original Spanish version of the questionnaire can be found in Questionnaire S1. To determine personality traits, Úbeda & Llorente (2015) conducted two different factorial analyses, the Principal Component Analysis (PCA) and the Regularized Exploratory Factor Analysis (REFA). Both methodologies determined the same personality dimensions or traits: Extraversion, Neuropsychoticism and Dominance. The trait Neuropsychoticism was a compound factor which included aspects of both Neuroticism and Psychoticism as described in PEN model for humans (Eysenck & Eysenck, 1964).

The four chimpanzees which were not included in the original study were assessed in March 2018 with the same questionnaire, filled by15 raters (26.67% men and 73.33% women). All raters were highly familiar with the subjects, as they all worked as researchers, volunteers or keepers and knew the animals for a minimum of 4 months. When raters did not answer a question, missing data on the ratings was substituted by a neutral score of 4 (Costa & McCrae, 2008; Weiss et al., 2009). Following the methodology of previous studies (Úbeda & Llorente, 2015; Weiss et al., 2009), inter-rater reliability was assessed by calculating two intraclass correlation coefficients (ICC) (Shrout & Fleiss, 1979) using IBM® SPSS® Statistics 22: ICC (3,1), which indicates the reliability of the scores for a single rater, and ICC (3, k), which indicates the reliabilities of scores based on the mean of the total number of raters. As described by Weiss et al. (2009), individual scores on each personality trait for all the 14 chimpanzees were obtained by summing unit-weighted scores of all the adjectives that had salient loadings (>0.50). We used the factor loadings derived from REFA analysis, as this methodology is specifically designed for small samples (Jung & Lee, 2011) (see Table 1).

**Table 1:** Factor loadings obtained for the eysencks PEN model based on a Regularized Exploratory Factor Analysis (REFA) (adapted from Úbeda & Llorente, 2015).

	**Extraversion**	**Neuropsychoticism**	**Dominance**
Spontaneous	**.79**	.02	.08
Active	**.80**	.07	.11
Sad	**−.76**	.23	.03
Social	**.71**	−.10	.12
Creative	.37	.06	−.10
Aggressive	.08	**.82**	.12
Anxious	−.06	**.69**	−.08
Impulsive	.43	**.65**	−.03
Cruel	−.22	**.56**	−.04
Bad tempered	−.46	**.61**	.13
Dominant	.23	.07	**.97**
Fearful	−.46	.11	−.38

### Correlations between personality ratings and behavior

In line with previous studies (Pederson, King & Landau, 2005), to further validate the results obtained with the Eysenck questionnaire, we used Spearman correlations to link the personality ratings with behavioral observations conducted at Fundació Mona for a longitudinal study. We used data collected over a total period of 133 months, from April 2006 to September 2017. Over this 11-year observation period, there were several changes in the group composition to integrate new chimpanzees, transfer animals between groups for welfare reasons or due to the natural death of individuals. While acknowledging the effect of this and other temporal factors (e.g., age, changes in well-being, etc.) on the development of chimpanzees’ personality and on their behavior, by definition personality should be stable across time and contexts. Moreover, there is evidence that, despite gradual changes over time, specific personality traits remain fundamentally stable and can therefore be detected at different developmental stages (Weiss et al., 2017).

Behavioral data were collected using the scan sampling method with 2-minute intervals. Behaviors observed included solitary activities (i.e., abnormal, locomotion, feeding, manipulation, inactivity, self-directed, and other solitary), social interactions (i.e., grooming, agonistic dominance, agonistic submission, other agonistic, social play, sexual behavior, other affiliative, and social proximity), and interactions with humans (positive and negative). Details on the behavioral catalogue are described in Table S1. Additionally, to facilitate interpretation of the correlations between personality ratings and observational data, we created categories which clustered several behaviors. In particular, we defined total agonistic interactions as the combination of agonistic dominance, agonistic submission and other agonistic behaviors; and total affiliative interactions included grooming, social play, sexual behavior and other affiliative behaviors.

We conducted observation sessions of 20 min from two observation towers in the outdoor enclosures. The sessions were randomly distributed during daytime hours, from 10:00 h to 18:30 h. Observations were conducted by different observers, who were only allowed to collect data after completing a training period and successfully passing the inter-observer reliability test (agreement between observers ≥ 85%).We included a total of 274204 scans (mean number of scans per individual ± SD = 19,586 ± 8348.21), resulting in 194,238 recorded behaviors, excluding the categories “not visible” and “not present” (15.55% and 13.59% respectively). We also excluded two additional categories from the analysis (“other social” and “other human interactions”) because they had very low frequencies (less than 0.02% of the scans). Due to the fact that not all chimpanzees were present from the beginning of the data collection period, data was normalized by calculating the relative frequencies of behaviors with respect to the total number of observed behaviors per individual.

### Experimental tasks and procedure

Cognitive tasks and experimental design are detailed in Riba (2016). In brief, the tasks consisted of 11 puzzle boxes made of methacrylate which included different components such as doors, wooden bars, slides and tubes (see Fig. S1). These elements needed to be manipulated in a particular manner for the chimpanzee to complete the task and obtain the food reward in the box (see details in Table 2). Tasks were classified based on their level of complexity (4 simple, 4 intermediate and 3 complex tasks), measured by means of the number of motor actions necessary to solve them. Thus, simple tasks were described as tasks which could be solved by performing a single motor action, intermediate tasks corresponded to those which required two motor actions, and complex tasks required the chimpanzees to perform three or more motor actions. The chimpanzees were assessed during a total period of 3 years and 7 months, between October 2009 and April 2013. One chimpanzee (Cheetah) had not yet arrived at the sanctuary at that time, and therefore she did not participate in the testing sessions.

**Table 2:** Overview of the experimental tasks classified by the level of complexity and description of the actions required to complete each task.

**Complexity level**	**Task**	**Actions required**
**Simple**	Open Box	(1) Pull/push the front door of the box
		Moveable Tube	(1) Pull/rotate a vertical moveable tube
		Windows Task	(1) Slide a horizontal wooden bar inserted in a tube
	Tube Cube	(1) Slide/rotate a horizontal tube inserted in the box
**Intermediate**	Artificial Fruit	(1) Push/pull a wooden bar
			(2) Slide a lid on the top
		Food Box	(1) Slide the frontal door
			(2) Insert a tool
		Push Box	(1) Slide a horizontal wooden bar
			(2) Push/pull the frontal door and insert a tool
		Tower Task	(1) Slide a horizontal wooden bar
	(2) Rotate/pull a vertical tube.	
**Complex**	Complex Food Box	(1) Slide 2 horizontal wooden bars (right side)
				(2) Slide 1 vertical wooden bar
				(3) Slide 2 horizontal wooden bars (left side)
			(4) Open a door on the top
		Complex Moveable Tube	(1) Slide 3 small wooden bars
				(2) Slide a large horizontal bar
				(3) Rotate/pull a tube
			(4) Slide the front door
		Complex Artificial Fruit	(1) Push/pull a wooden bar (right side)
				(2) Pull/push a wooden bar (central position)
				(3) Pull/push a wooden bar (left side)
				(4) Slide a lid on the top
	(5) Rotate a plastic tube	

The first 5 months corresponded to a pilot phase of the study in which all subjects were exposed to three random tasks in three different sessions. To do so, the chimpanzees were isolated by one familiar keeper in an area called the exterior cages (see description above), where all subsequent experimental sessions were conducted. These habituation sessions lasted 10 min and the chimpanzees could see the puzzle boxes, which were placed within sight outside the cages, but they were not allowed to interact with them. The objectives of the pilot phase were to (1) habituate the subjects to the study area and to the cart which would support the puzzle boxes, (2) train the keepers who were going to participate and/or be present during the experimental sessions, and (3) check for intrinsic aspects of the tasks and the procedure, such as the position of the device on the cart or the type of fixation. After this pilot phase, the animals were presented first with simple tasks (2010–2011), followed by intermediate tasks (2011–2012), and finally complex tasks (2012–2013). Tasks never overlapped in time, and each of them was presented separately within a period of two to three months. The experimental sessions were randomly distributed throughout this period, according to the keepers’ availability and other management needs.

The original purpose of the study by Riba (2016) was to investigate the occurrence of social learning. Therefore, before being tested, subjects received three different types of information on how to solve the puzzle boxes: (1) Control (no information), (2) No social information (the subject only saw the end state of the task, without seeing any of the actions necessary to solve it), and (3) Social information (the subject saw both the actions of the demonstrator and the end state of the task). Each subject received all three conditions in each set of complexity level, but subjects were exposed to a different task within each complexity level. Thus, task, level of complexity and type of information were counterbalanced across subjects, resulting in a total of nine possible combinations This means that not all subjects performed all the tasks, but they all performed 3 simple, 3 intermediate and 3 complex tasks (one Control, one with No social information and one with Social information for each complexity level; see Table S2). Additionally, to evaluate the effect of causal information, every task consisted in two versions of the puzzle box, one transparent and one opaque. Therefore, for each task, subjects were exposed to both the transparent and the opaque version of the apparatus, within the same session. The order in which the two versions were presented was counterbalanced between subjects.

**Figure 1:**
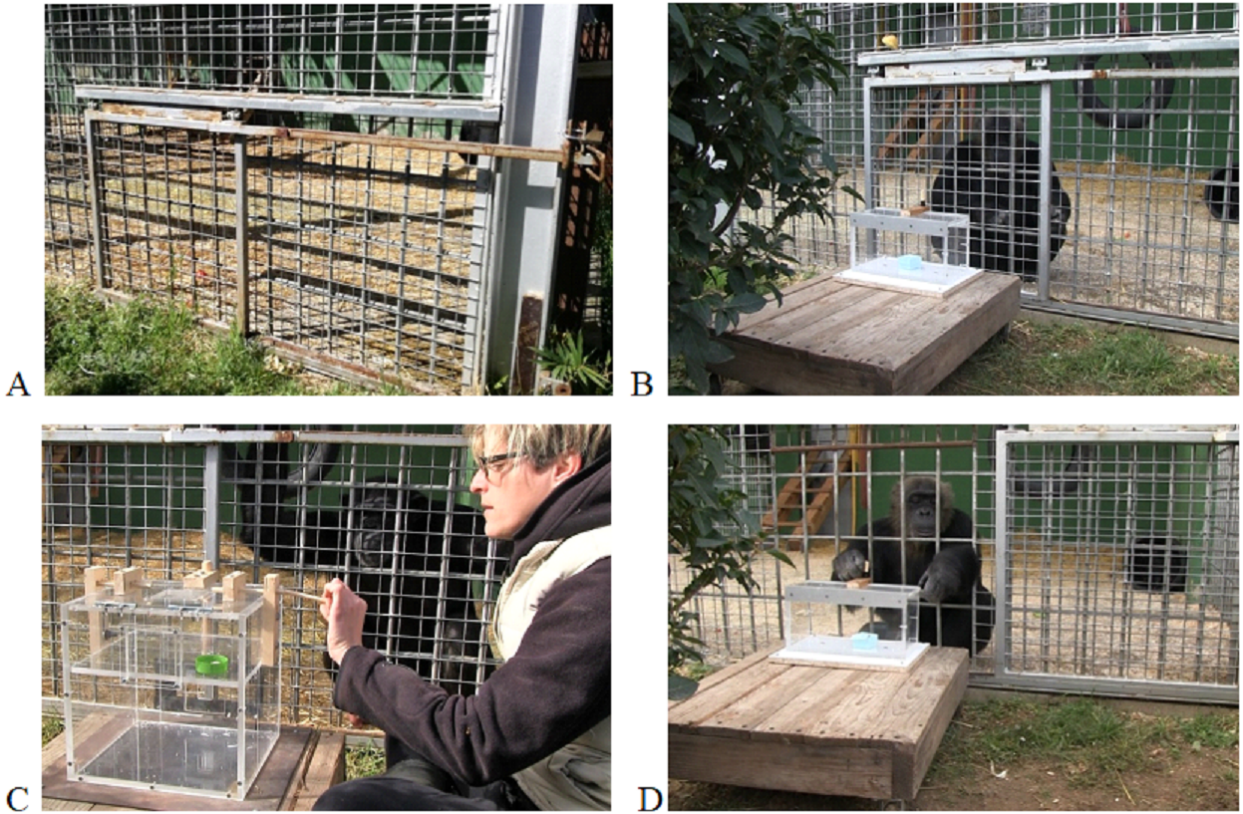
Exterior cages in which the experimental sessions were conducted. (A) Detail of the sliding door. (B) Exposure phase: the door was closed, so that the chimpanzee could see but not touch the apparatus. (C) Social information condition: a keeper performed the task in front of the individual. (D) Testing phase: the sliding door had been displaced and the subject could interact with the task through the bars.

The experimental session began when the subject was called by a keeper to participate in the experiment and entered the exterior cages. After this, the door to the indoor facilities was closed and the subject remained isolated from the group. Before starting the testing phase, there was a 10-minute habituation phase, in which the keeper stayed in close proximity to the cages and the chimpanzees could already see the experimenter and the apparatus from afar (in its initial state). During this phase, the experimenter conducted *ad libitum* observations, particularly looking for behavioral signs of anxiety or discomfort (e.g., abnormal or stereotypical behaviors, agonistic displays). When these behaviors were detected and the chimpanzee did not approach the apparatus in the first 5 min, the session was terminated. Conversely, if no signs of distress were observed during the habituation phase, the experimenter placed the apparatus in front of the subject, specifically in front the barred sliding door of the exterior cages, through which the animals could see it but not touch it (Figs. 1A and 1B). This allowed the subjects to familiarize with the puzzle boxes before starting the testing phase. Exposure time varied according to task complexity (i.e., 1 min for simple tasks, 2 min for intermediate tasks and 3 min for complex tasks). Additionally, in the No social information condition, the apparatus was removed from the individual’s view after the first exposure, so that the human experimenter could manipulate it. Then, it was presented once again to the subject in its final state (solved), with the sliding door remaining closed, thus preventing the chimpanzee to reach the apparatus. Exposure time to this final state also varied according to task complexity (6 min for simple tasks, 12 min for intermediate tasks and 20 min for complex tasks). The time elapsed between the two types of exposure (initial state and final state of the apparatus) was between 2 and 5 min, depending on the task complexity. Afterwards, the apparatus was moved out of the chimpanzees’ view, so that the experimenter could return it to its initial state (not solved). Immediately after that, it was placed again in front of the animal, before starting the testing phase. Finally, in the Social information condition, the experimenter performed the task in front of the individual for several times (6 demonstrations for simple tasks, 12 demonstrations for intermediate tasks and 20 demonstrations for complex tasks) with the apparatus facing the animal (Fig. 1C).

In all conditions, the testing phase began when the sliding door was displaced (Fig. 1D), thus allowing subjects the first contact with the task. The chimpanzees were presented with one puzzle box per session and they had 8 attempts to solve it (4 for the transparent version and 4 for the opaque version). The time allowed for the solution of the task was 2 min for the simple tasks, 4 min for the intermediate tasks and 6 min for the complex tasks. After this time had elapsed, the trial ended and the apparatus was moved out of the individual’s reach, so that the experimenter could return it to its initial state. Immediately after that, it was presented to the subject again, thus initiating a new trial. In total, each subject participated in 9 experimental sessions of 8 trials each, thus making a total of 72 trials: 24 trials for the simple tasks, 24 for the intermediate tasks and 24 for the complex tasks (see details in Table S2). For two chimpanzees (Juanito and Tom) a few trials in the complex tasks (9 and 8, respectively) had to be discarded due to demonstration failures and camera failure. Besides the experimenter, one familiar keeper was always present throughout the habituation and the testing phases, to provide animals with a safe trusted environment during the tasks. All sessions were videotaped for subsequent analyses with a digital camera placed in a frontal or a semi-lateral position at a distance of 60–80 cm from the subject. Video coding of all sessions was conducted by a single experimenter (David Riba).

### Performance measures

We used participation as a measure of interest, success and latency to solve the task as measures of performance, and losing contact with the task as an indicator of lack of motivation. When chimpanzees (1) refused to enter the experimental area, (2) did not approach the apparatus or (3) did not establish contact with it, we assigned them a score of 0 for participation. On the other hand, if they interacted with the apparatus, even if it was for a very short time, we assigned them a score of 1. An attempt was considered successful if the subject completed the task and retrieved the reward from the box within the given time. Latency was described as the time (in seconds) between the first contact with the apparatus and the moment the task was solved. Finally, we considered that a subject lost contact with the task if it stopped manipulating the apparatus or its components for more than 15 s and/or walked away at least 1 meter. We only considered the first-time that subjects lost contact. Thus, for each trial, a subject was assigned a score of 0 if it remained engaged with the task the whole time and a score of 1 if it stopped manipulating it at least once.

### Data analysis

To investigate whether interest, motivation and performance were affected by personality, we used Generalized Linear Mixed Models (GLMM) (Baayen, 2008). For response variables with binomial distribution (participation, success and losing contact with the task), we used the function “glmer” from the package “lme4” (version 1.1-17; Bates et al., 2015) in R (R Core Team, version 3.5.0), whereas for latency, which had a normal distribution, we used the “lmer” function.

We ran 4 different models, one for each response measure as dependent variable: participation (Model 1), success (Model 2), latency (Model 3) and losing contact with the task (Model 4). In all models, age and personality traits (Extraversion, Neuropsychoticism and Dominance) in interaction with sex were included as test predictors, whereas we entered task complexity (simple, intermediate or complex), information provided to the subject (Control, No social information or Social information), trial number (1–4), box color (opaque or transparent) and box order (first opaque or first transparent) as control predictors. We included subject’s identity as random effect, fitting random slopes as needed.

In all models, continuous predictors were *z*-transformed to facilitate model convergence and standardize interpretation of model coefficients. To compare full models containing all predictors with null models containing only control predictors, we used a likelihood ratio test (function “anova”) (Dobson, 2002). If full models significantly differed from null models (*p* ≤ 0.05), we conducted likelihood ratio tests to obtain the *p* values for each predictor via single-term deletion, using the R function drop1 (Barr et al., 2013). If the 2-way interactions were not significant, we downgraded them and re-ran the model including the 2 test predictors as main effects. To rule out collinearity, we calculated variance inflation factors (VIF) (Field, 2009), which were very good in all models (maximum VIF across models = 2.39). Finally, we assessed dispersion for the non-gaussian models and we found that, none of them was over-dispersed (dispersion parameters <1), except for Model 4. Therefore, in order to avoid over-dispersion, we ran a simplified version of the model, removing the control predictors “task complexity” and “information type”, as well as the random slopes. No convergence issues were detected in the models.

### Ethics statement

All applicable international, national, and/or institutional guidelines for the care and use of animals were followed. All procedures involving animals were in accordance with the ethical standards of the institution at which the studies were conducted (Fundació Mona; Ethical Approval Number: EAFM201801) and with the Spanish Government RD 53/2013. This project also received the ethical approval from the Ethics Committee of the Universitat de Girona (Project Code: CEBRU0020-2019).

## Results

### Personality ratings

Considering the 14 chimpanzees whose personality was assessed between 2012 and 2018, the ICCs for the single (3, 1) and average (3, k) ratings were high, indicating that raters tended to agree in their judgments about the personality items (Table 3). ICC (3, 1) ranged from 0.19 (*creative*) to 0.50 (*aggressive*), with a mean reliability of 0.38. On the other hand, ICC (3, k) ranged from 0.59 (*creative*) to 0.86 (*aggressive*), with a mean reliability of 0.77. After being transformed into T-scores (mean ± SD = 50 ± 10), the values of the personality traits ranged from 22.02 to 63.44 (Extraversion), from 35.17 to 64.84 (Neuropsychoticism), and from 37.90 to 63.52 (Dominance).

**Table 3:** Intraclass correlation coefficients (ICCs) for the 12 items of the questionnaire. ICC (3, 1) indicates the reliability of the scores for a single rater, and ICC (3, *k*) indicates the reliabilities of scores based on the mean of the total number of raters.

	**ICC (3, 1)**	**ICC (3, k)**
Social	.42	.81
Active	.49	.85
Dominant	.44	.82
Spontaneous	.38	.79
Anxious	.38	.78
Badtempered	.37	.78
Fearful	.29	.71
Sad	.37	.78
Agresssive	.50	.86
Impulsive	.40	.80
Cruel	.28	.70
Creative	.19	.59
**Mean**	**.38**	**.77**
**SD**	**.09**	**.07**

### Correlations between personality ratings and behavior

We obtained significant correlations between personality traits and behaviors which matched their descriptions (Table 4 and Table S3). In particular, Extraversion was positively correlated with social behaviors (i.e., grooming, social play, and the combined category total affiliative interactions); Neuropsychoticism was positively associated with total agonistic interactions (which included both dominant and submissive behaviors); and Dominance positively correlated with agonistic dominance, but also with total agonistic interactions. We also found unexpected correlations, such as higher Extraversion being linked to agonistic dominance and Neuropsychoticism being negatively associated with foraging. Moreover, contrary to our predictions, Neuropsychoticism was not related to behavioral indicators of anxiety, such as self-directed behaviors or abnormal behaviors. Finally, regarding the associations between traits, no significant correlations were found, but the positive correlation between Dominance and Neuropsychoticism was close to significance (Table 5).

**Table 4:** Behaviors and clusters of behaviors that correlated (Spearman correlation) with Eysencks personality traits.

		**Agonistic dominance**	**Grooming**	**Social play**	**Foraging**	**Agonistic interactions**^a^	**Affiliative interactions**^b^
**Extraversion**	***r***	**.614**	**.705**	**.692**	.147	.529	**.730**
	***p***	**.020**	**.005**	**.006**	.615	.052	**.003**
	**95% CI**	**[.079, .929]**	**[.147, .957]**	**[.250, .928]**	[−.385, .668]	[−.019, .862]	**[.355, .888]**
**Neuropsychoticism**	***r***	.211	.099	−.115	**−.640**	**.562**	.064
	***p***	.469	.737	0.697	**.014**	**.037**	.828
	**95% CI**	[−.323, .697]	[−.408, .557]	[−.653, .596]	**[−.945, −.172]**	**[.137, .806]**	[−.493, .554]
**Dominance**	***r***	**.557**	.547	−.084	−.055	**.594**	.378
	***p***	**.039**	.043	.776	.852	**.025**	.182
	**95% CI**	**[.028, .892]**	[−.014, .900]	[−.623, .454]	[−.563, .476]	**[.118, .871]**	[−.236, .811]

**Notes.**

*N* = 14. Significant results are marked in bold (*p* < 0.05; 95% CI do not overlap 0).

a Agonistic interactions included agonistic dominance, agonistic submission and other agonistic behaviors.

b Affiliative interactions included grooming, social play, sexual behavior and other affiliative behaviors.

**Table 5:** Spearman correlations between chimpanzees’ scores on Eysenck’s personality dimensions.

		**Extraversion**	**Neuropsychoticism**
**Extraversion**	***r***		
	***p***	**–**	**–**
	**95% CI**		
**Neuropsychoticism**	***r***	.073	
	***p***	.805	**–**
	**95% CI**	[−.385, .470]	
**Dominance**	***r***	.429	.525
	***p***	.126	.054
	**95% CI**	[−.220, .886]	[−.115, .865]

**Notes.**

*N* = 14. Significant results are marked in bold (*p* < 0.05; 95% CI do not overlap 0).

### Association between personality traits and interest, motivation and performance

The results obtained in the cognitive tasks are summarized in Table S4. Participation and success were high (mean participation ± SD = 0.81 ± 0.22, range = 0.35–1.00; mean success ± SD = 0.91 ± 0.13, range = 0.57–1.00) and the chimpanzees lost contact with the task very rarely (mean value of losing contact with the task ± SD = 5.21 ± 8.36%, range = 0.00–21.00). Mean latency across all tasks ± SD = 30.55 ± 15.57 s and, as expected, it differed significantly across complexity levels (*χ*^2^ = 18.00, *df* = 2, *p* < 0.001). Results of the 4 models evaluating the relationship between personality and participation, success, latency and losing contact with the task are presented in Table 6.

*Participation*. In Model 1, the full model significantly differed from the null model (GLMM: *χ*^2^ = 26.98, *df* = 8, *p* < 0.001), but none of the test predictors had a significant effect. After downgrading the non-significant 2-way interactions and including personality traits and sex as main effects, the full-null model comparison was not significant, thus revealing that none of the test predictors predicted participation.

**Figure 2:**
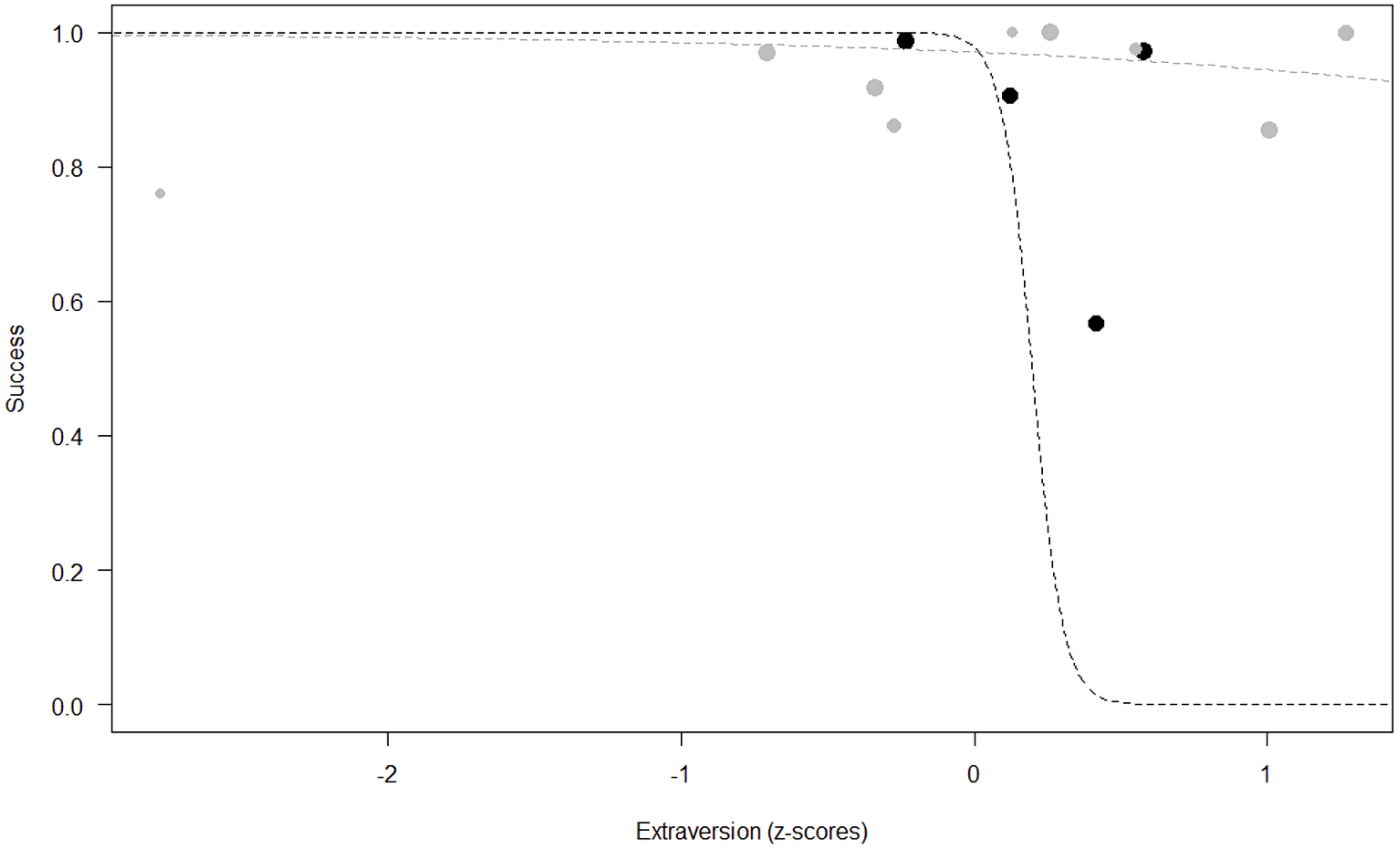
Probability of success as a function of Extraversion. The dots represent the individuals tested (females in black, males in grey), with their size being proportional to the number of trials in which they participated. The dashed lines depict the models, which have been back-transformed from the log-odds ratio scale (black for females, grey for males).

**Figure 3:**
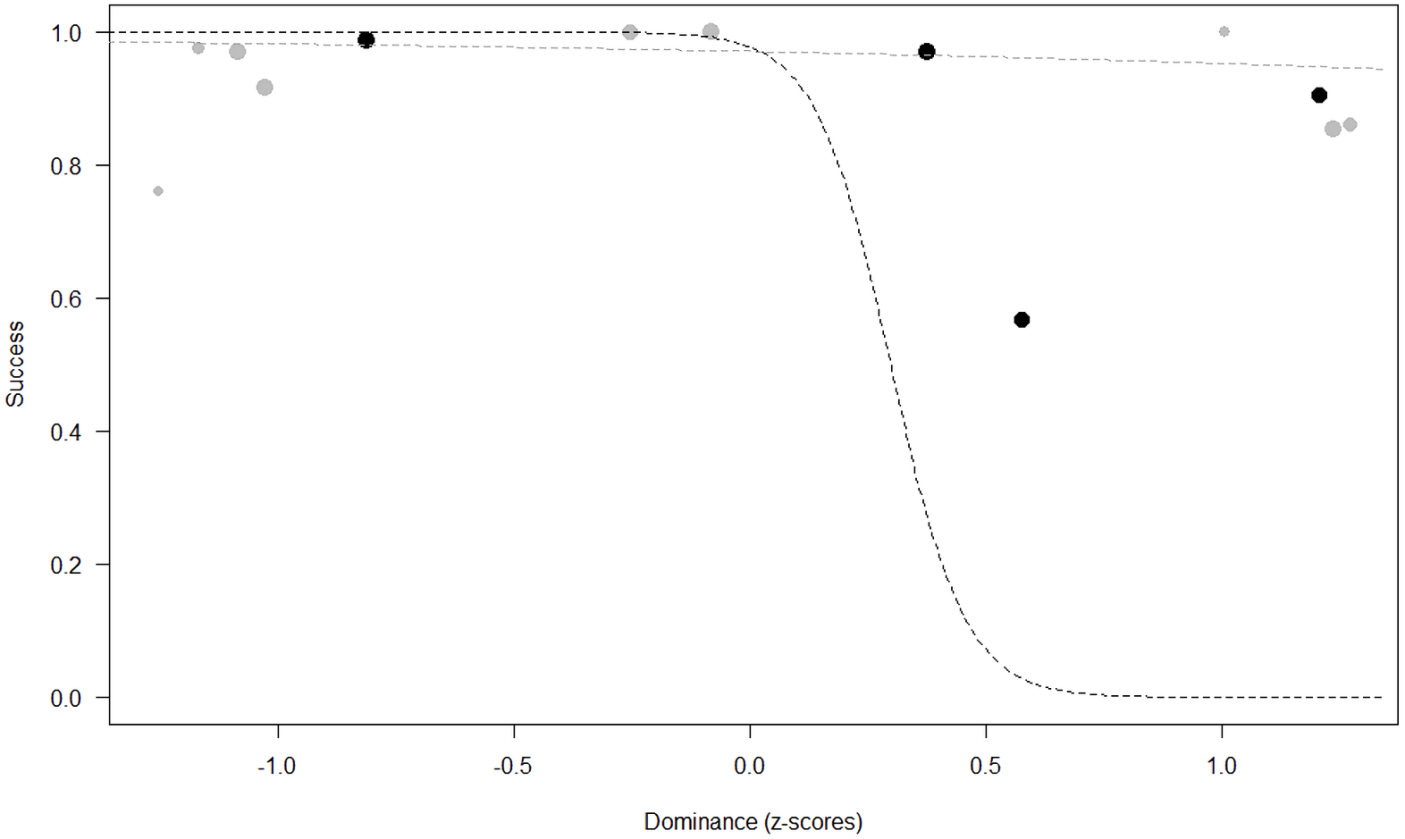
Probability of success as a function of Dominance. The dots represent the individuals tested (females in black, males in grey), with their size being proportional to the number of trials in which they participated. The dashed lines depict the models, which have been back-transformed from the log-odds ratio scale (black for females, grey for males).

**Figure 4:**
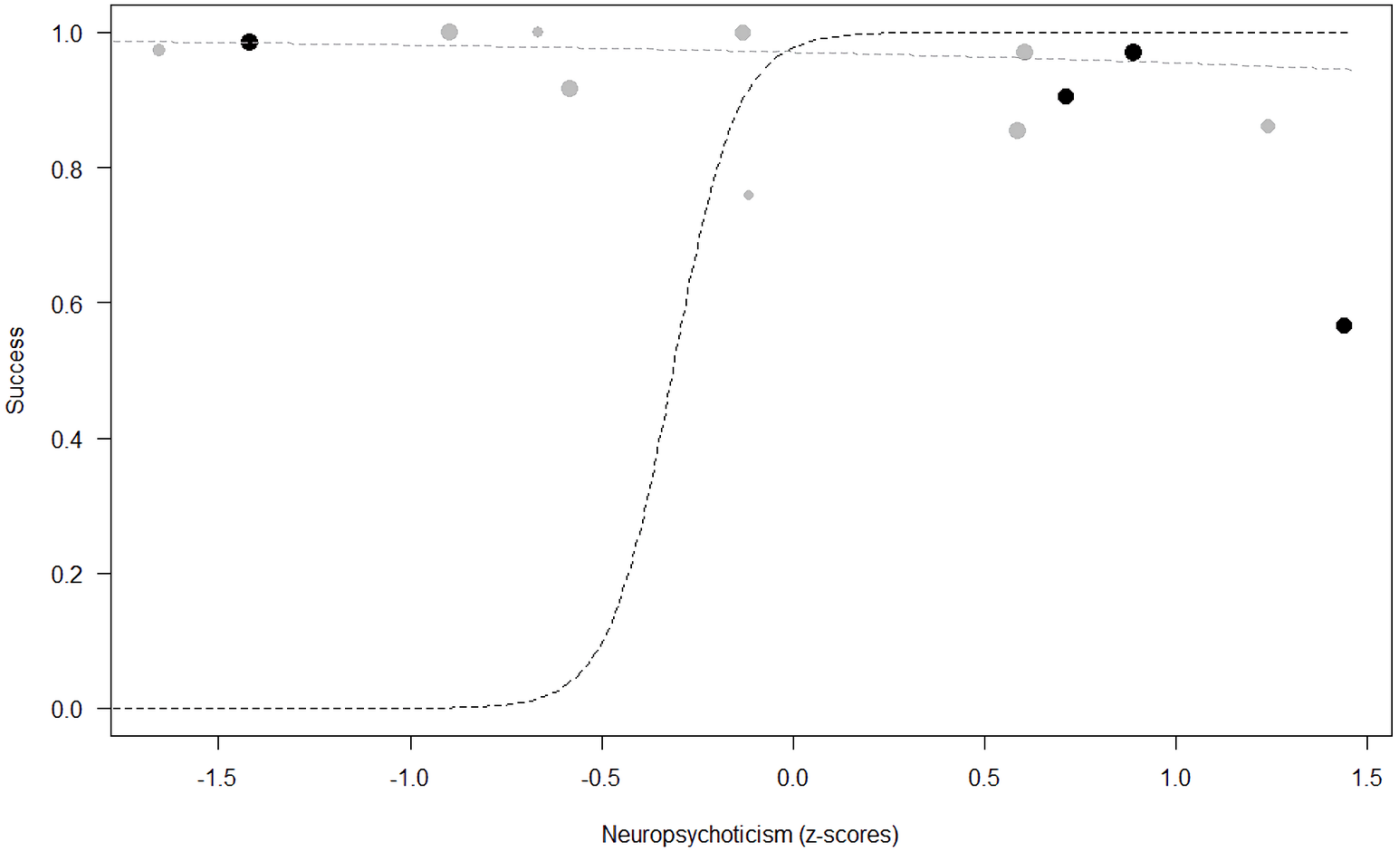
Probability of success as a function of Neuropsychoticism. The dots represent the individuals tested (females in black, males in grey), with their size being proportional to the number of trials in which they participated. The dashed lines depict the models, which have been back-transformed from the log-odds ratio scale (black for females, grey for males).

*Success.* In Model 2, the comparison between full and null models was significant (GLMM: *χ*^2^ = 15.84, *df* = 8, *p* = 0.045). All personality traits in interaction with sex predicted chimpanzees’ success (Extraversion*sex: *p* = 0.012; Neuropsychoticism*sex: *p* = 0.003; Dominance*sex: *p* < 0.001), but the test predictor age was not significant. In particular, lower Extraversion slightly increased the probability of being successful in males, while highly increasing it in females (see Table 6; Fig. 2). Similarly, lower Dominance predicted a higher probability of success in both sexes, but this effect was stronger in females (see Table 6; Fig. 3). Finally, higher Neuropsychoticism predicted a higher probability of female success, but a slightly lower probability of success in males (see Table 6; Fig. 4).

*Latency.* In Model 3, the comparison between the full and null model was not significant (GLMM: *χ*^2^ = 2.37, *df* = 8, *p* = 0.967), even after downgrading the 2-way interactions and re-running the model including the personality traits and sex as main effects. Thus, personality traits, sex and age did not predict individuals’ latency to complete the task.

**Figure 5:**
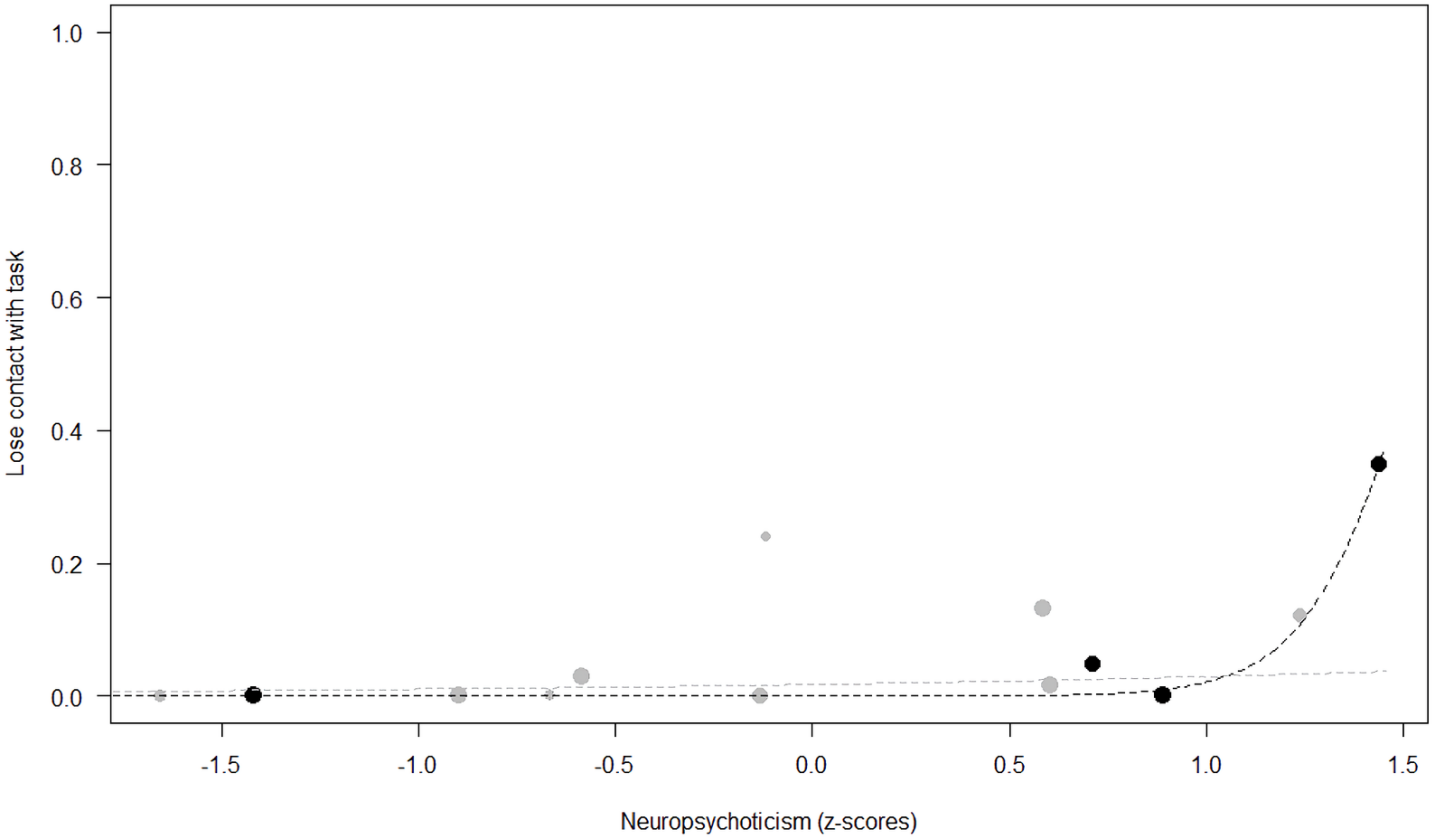
Probability of losing contact with the task as a function of Neuropsychoticism. The dots represent the individuals tested (females in black, males in grey), with their size being proportional to the number of trials in which they participated. The dashed lines depict the models, which have been back-transformed from the log-odds ratio scale (black for females, grey for males).

**Figure 6:**
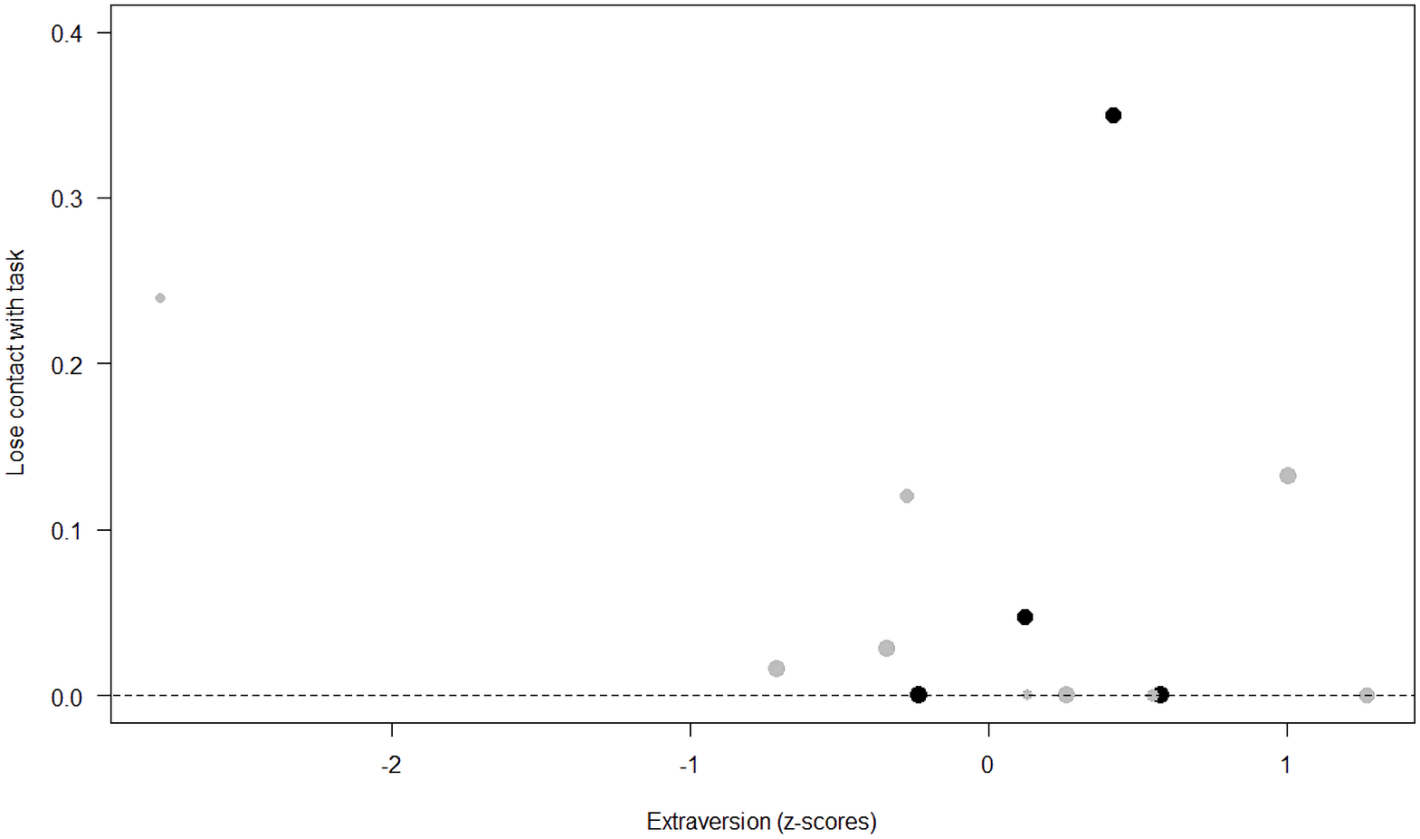
Probability of losing contact with the task as a function of Extraversion. The dots represent the individuals tested (females in black, males in grey), with their size being proportional to the number of trials in which they participated. The dashed lines depict the model, which has been back-transformed from the log-odds ratio scale.

**Figure 7:**
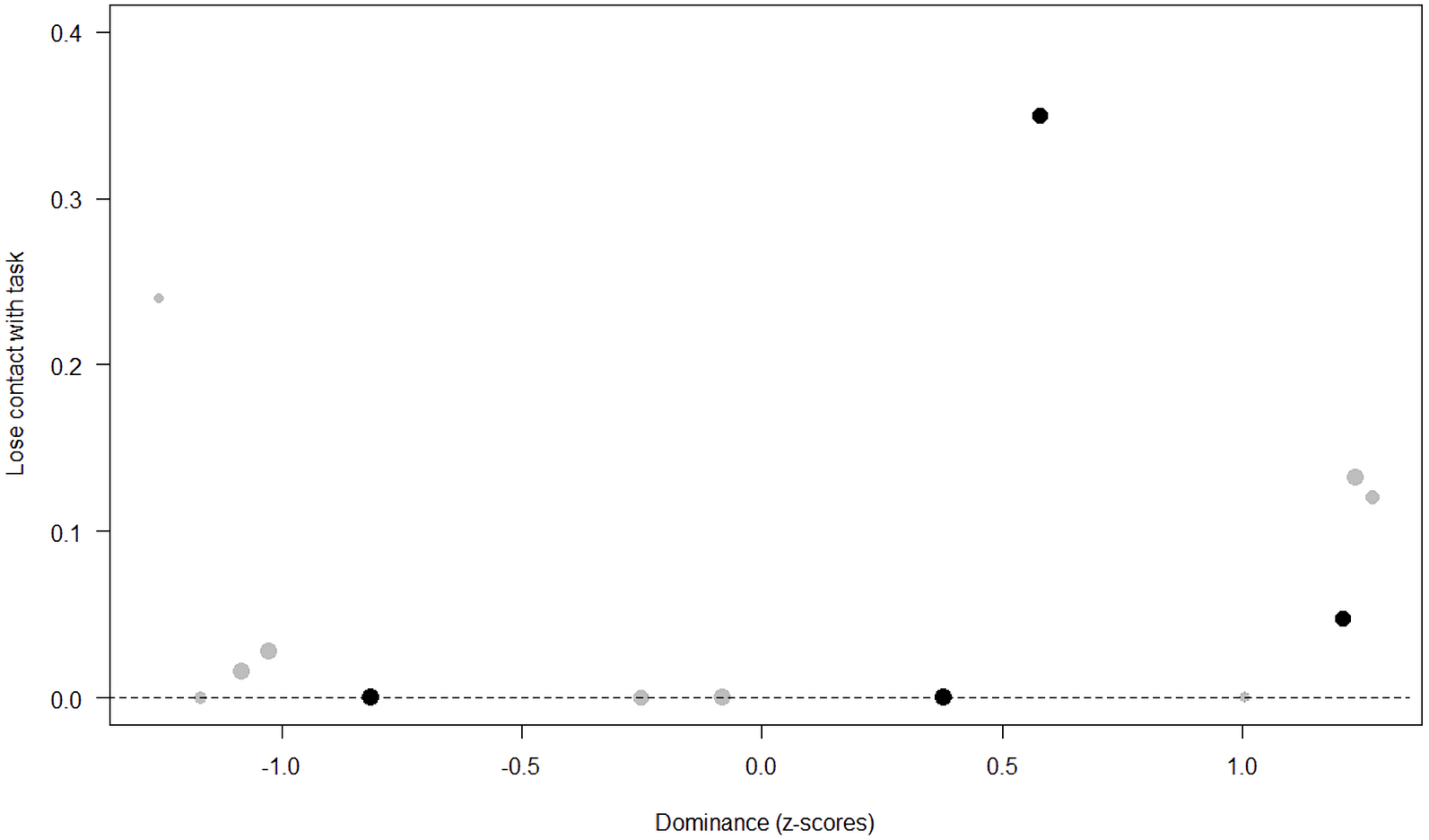
Probability of losing contact with the task as a function of Dominance. The dots represent the individuals tested (females in black, males in grey), with their size being proportional to the number of trials in which they participated. The dashed lines depict the model, which has been back-transformed from the log-odds ratio scale.

**Figure 8:**
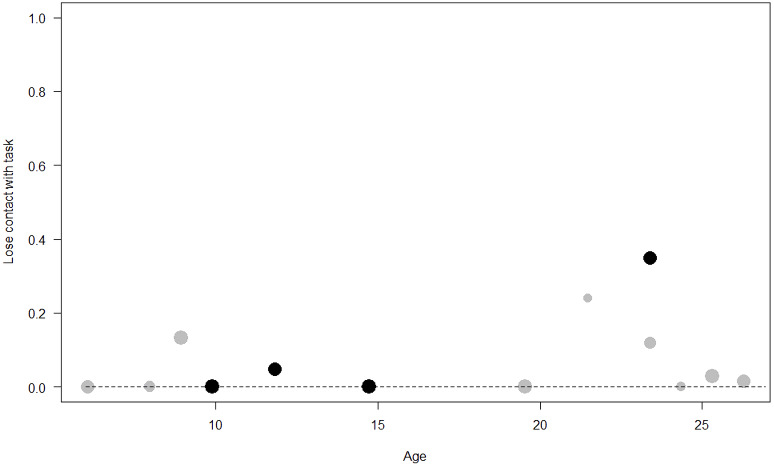
Probability of losing contact with the task as a function of age. The dots represent the individuals tested (females in black, males in grey), with their size being proportional to the number of trials in which they participated. The dashed lines depict the model, which has been back-transformed from the log-odds ratio scale.

*Losing contact with the task.* In Model 4, the full-null model comparison was significant (GLMM: *χ*^2^ = 27.48, *df* = 8, *p* < 0.001). Neuropsychoticism in interaction with sex and age were the only significant predictors of the probability of losing motivation and stopping manipulation of the task. After downgrading the non-significant 2-way interactions, we also found a significant effect of Extraversion (*p* < 0.001), Dominance (*p* = 0.012), age (*p* = 0.019), and the 2-way interaction of Neuropsychoticism and sex (*p* = 0.002). In particular, higher Neuropsychoticism highly increased the probability of losing contact with the task in females, and only slightly increased it in males (see Table 6; Fig. 5). In both sexes, higher Extraversion was linked to a lower probability of losing motivation and stopping manipulation of the task (see Table 6; Fig. 6), whereas higher Dominance was associated with a higher probability of losing contact with the task in both sexes (see Table 6; Fig. 7). Finally, younger individuals had a higher probability to lose motivation and stop interacting with the task, as compared to older ones (see Table 6; Fig. 8).

## Discussion

In this study, we first compared behavioral observations of 14 captive chimpanzees with ratings from a 12-item personality questionnaire based on Eysenck’s PEN model (Úbeda & Llorente, 2015); and then we assessed the relationship between personality traits and interest, motivation and performance in cognitive tasks in a subsample of 13 individuals. Firstly, the traits obtained from the ratings significantly correlated with behavioral observations conducted over an 11-year period, but some expected correlations were absent. Secondly, our results showed that participation and latency were not associated with any personality trait from the PEN model. Partially in line with our predictions, the probability of success increased with lower Extraversion and lower Dominance, but this was more evident for females. Unexpectedly, success was also higher in females with higher Neuropsychoticism. The probability of losing motivation and stopping interaction with the task were higher in younger chimpanzees, and in those rated higher on Dominance and lower on Extraversion. Finally, and in agreement with our predictions, individuals scoring higher in Neuropsychoticism were also more likely to lose motivation, especially in females.

Inter-rater reliabilities in the personality questionnaires were similar to those reported in previous studies (Úbeda & Llorente, 2015; King & Figueredo, 1997; Weiss et al., 2009), and indicated substantial agreement among raters. The correlations between personality traits and behaviors confirmed that there was some evidence for convergent validity. In particular, Extraversion positively correlated with total affiliative behaviors and with grooming and social play considered separately; and Dominance correlated with total agonistic interactions and with agonistic dominance independently. These associations were similar to those reported in previous studies on chimpanzees (Pederson, King & Landau, 2005; Vazire et al., 2007) and other great apes (Eckardt et al., 2015; Kuhar et al., 2006; Schaefer & Steklis, 2014). Moreover, Neuropsychoticism positively correlated with total agonistic interactions, confirming that chimpanzees with higher Neuropsychoticism are in fact more anxious, impulsive and aggressive, which is also consistent with Eysenck’s definition of these traits (Eysenck & Eysenck, 1964). Neuropsychoticism was also negatively associated with foraging, which we interpreted as neuropsychotic chimpanzees being less prone to explore the enclosures to look for food, or perhaps dedicating more time to vigilance (Digman, 1990) or to aggressive interactions. Another possible explanation could be that neuropsychotic individuals have reduced levels of activity, as it has been found in bonobos that show more anxious behavior (i.e., higher rates of self-scratching) (Staes et al., 2016). Hence, a decrease in foraging would simply be a consequence of lower levels of general activity. Contrary to our predictions, however, Neuropsychoticism was not related to behavioral indicators of anxiety, such as self-directed behaviors or abnormal behaviors. However, it should be noted that our definition of self-directed behaviors included some behaviors, such as body inspection and self-grooming, which may not necessarily be indicators of anxiety or stress (Meyer & Hamel, 2014). Finally, we found some unexpected correlations, such as higher Extraversion being linked to agonistic dominance. Surprisingly, previous studies have reported an association between aggression and Extraversion in chimpanzees (Freeman et al., 2013) and in gorillas (Kuhar et al., 2006). Nonetheless, an important limitation of our study is that the category agonistic dominance encompassed a wide range of behaviors, from directed displays to resource displacement, but also aggression. Therefore, to further investigate the association between personality and aggression, a more detailed behavioral catalogue should be employed in the future, to better distinguish between aggressive and non-aggressive dominant behaviors.

**Table 6:** Results of Models 1–4. For each model and predictor, estimates, standard errors (SE), likelihood ratio tests (LRT), degrees of freedom (df), and *p*-values (*p*).

**Models**	**Estimate**	**SE**	**LRT**	**df**	***P***
**Model 1: Participation**					
Intercept	1.151	1.794	–	–	–
Dominance	−1.320	1.578	0.84	1	0.360
Extraversion	2.383	1.240	3.51	1	0.061
Neuropsychoticism	−1.025	1.582	0.55	1	0.458
Sex (male)	4.486	2.179	3.81	1	0.051
Age	1.477	1.486	1.18	1	0.278
Task complexity	−0.256	0.564	0.21	1	0.648
Information type	0.747	0.654	1.43	1	0.232
Trial number	−0.297	0.138	4.53	1	**0.033**
Box color	0.328	0.285	1.28	1	0.258
Box order	−0.470	0.396	1.36	1	0.244
**Model 2: Success**					
Intercept	6.934	2.282	–	–	–
Dominance	−13.120	2.906	–	–	–
Extraversion	−23.638	7.868	–	–	–
Neuropsychoticism	12.106	3.374	–	–	–
Sex (male)	−3.328	2.191	–	–	–
Dominance*Sex(male)	12.676	3.042	11.03	1	**<0.001**
Extraversion*Sex(male)	22.811	7.642	6.28	1	**0.012**
Neuropsychoticism*Sex(male)	−12.563	3.462	8.78	1	**0.003**
Age	−1.226	0.819	1.95	1	0.162
Task complexity	−0.290	0.606	0.19	1	0.660
Information type	0.977	0.252	12.17	1	**<0.001**
Trial number	0.805	0.191	20.51	1	**<0.001**
Box color	0.130	0.358	0.13	1	0.719
Box order	−0.205	0.412	0.24	1	0.623
**Model 3: Latency**					
Intercept	46.724	9.043	–	–	–
Dominance	−1.123	3.697	0.19	1	0.667
Extraversion	4.230	4.150	1.70	1	0.193
Neuropsychoticism	0.671	3.375	0.06	1	0.800
Sex (male)	7.176	5.838	2.36	1	0.125
Age	0.677	3.364	0.11	1	0.742
Task complexity	41.209	1.983	339.59	1	**<0.001**
Information type	−2.465	1.872	1.72	1	0.190
Trial number	−5.545	1.533	13.16	1	**<0.001**
Box color	0.819	3.134	0.08	1	0.781
Box order	1.682	3.298	0.18	1	0.669
**Model 4: Lose contact with task**					
Intercept	−11.514	1.867	–	–	–
Dominance	1.033	0.451	6.35	1	**0.012**
Extraversion	−1.466	0.340	12.50	1	**<0.001**
Neuropsychoticism	7.433	1.337	–	–	–
Sex (male)	7.572	1.687	–	–	–
Dominance*Sex(male)	–	–	–	–	–
Extraversion*Sex(male)	–	–	–	–	–
Neuropsychoticism*Sex (male)	−6.901	1.434	10.02	1	**0.002**
Age	−0.930	0.337	5.52	1	**0.019**
Task complexity	–	–	–	–	–
Information type	–	–	–	–	–
Trial number	−0.451	0.176	6.91	1	**0.009**
Box color	0.168	0.345	0.24	1	0.626
Box order	0.123	0.354	0.12	1	0.727

**Notes.**

*N* = 13. Reference categories for categorical predictors are included in parentheses. Significant results are marked in bold. Personality traits, age and trial number were *z*-transformed prior to analyses. In all models, subject identity was included as random effect. In Model 4 complexity and information type were removed from the model to avoid overdispersion.

It should also be noted that some behaviors or clusters of behaviors correlated with more than one trait (e.g., agonistic dominance correlating with both Dominance and Extraversion; and total agonistic interactions correlating with both Dominance and Neuropsychoticism), thus revealing limited discriminant validity for the PEN model.

Nevertheless, this was not entirely unexpected. Firstly, the positive correlation between the traits Dominance and Neuropsychoticism, which was close to significance, suggested that, at least in our study sample, these two traits were partially associated. Therefore, it was no surprise that, some behaviors were common for both traits. Furthermore, finding a straightforward correspondence between personality traits and behaviors is a challenging endeavor (Pederson, King & Landau, 2005; Konečná et al., 2008), as several traits likely play a role in defining how a subject behaves (Capitanio, 2004). In particular, given that the questionnaire used in this study was fairly short, and only three dimensions were considered, the convergence of several behaviors in one trait was expected. Hierarchical personality models, like Eysenck’s or the FFM, describe higher order traits which include several specific traits; and these lower traits are characterized by several behavioral responses (Eysenck, 1990; DeYoung, 2006; DeYoung, 2010). Finally, it should be noted than we only had a small sample of chimpanzees, all coming from the same site (a primate rescue centre), with some of them having been exposed to traumatic past experiences which most likely shaped their personality (Ortín et al., 2019) and their behavior (Crailsheim et al., 2020).

Regarding our predictions for personality and performance in the puzzle boxes, only the models for success and losing contact with the task were significant. In view of the association between Extraversion in the PEN model and Openness in the FFM (Vorkapić, 2012); and taking into account that, by definition, extraverts are more explorative and curious, we expected individuals higher in Extraversion to be more interested in the tasks (i.e., to participate more). Additionally, considering the male-dominated hierarchy of chimpanzees both in the wild (Kaburu & Newton-Fisher, 2015; Newton-Fisher, 2004) and in captivity (De Waal, 1986; Noë, De Waal & Van Hooff, 1980), it would not be surprising that dominant males would feel more confident in front of a novel stimulus. Previous studies in chimpanzees reported a positive association between Dominance and participation in cognitive testing, but with inconsistencies across tasks (Altschul et al., 2017). Hopper et al. (2014) found that males scoring higher in Dominance spent more time interacting with a foraging puzzle, which can also be considered an indicator of interest. However, neither Extraversion nor Dominance was related to participation in the puzzle boxes. A possible explanation might be that, in our questionnaire, none of the adjectives directly assessed curiosity or exploration. Among the adjectives that loaded onto Extraversion, we find “spontaneous” and “active”, which could somehow be related to exploration, but we also have “not sad” and “social”, which may not be particularly relevant in a testing context. Therefore, Extraversion in our model may be more descriptive of the social aspect of the trait. Finally, it should be pointed out that, in this study, chimpanzees were actively encouraged to participate in the experimental sessions by the keepers. Therefore, we could assume that extraverts would show a greater response to this social stimulus, or rather that the role of the keeper might have affected the results by greatly increasing overall participation, regardless of personality. In the future, it would be recommended to set up an experimental design in which subjects can actively decide whether to engage in the task or not.

The probability to be successful was positively associated with lower Dominance and lower Extraversion in both sexes, although for both traits this relationship was more evident in females. This would contradict previous findings in chimpanzees indicating that dominant males were more successful in a foraging puzzle (Hopper et al., 2014). However, our results are in line with an experiment using a touchscreen testing system (Leighty et al., 2011), in which dominant mandrills (*Mandrillus sphinx*) required more sessions to be successful than subordinates. The authors suggested that dominant monkeys were more likely to focus their attention on social interactions than engaging in solitary activities, and were thus less successful. Similarly, Morton, Lee & Buchanan-Smith (2013) found a negative relationship between Assertiveness and performance in capuchin monkeys, which they also attributed to highly assertive individuals prioritizing social interactions over task engagement. In line with this, it has been stated that male chimpanzees would be primarily interested in social relationships and dominance hierarchy, as compared to females (Lonsdorf, 2005). However, in this study, we found no evidence that the link between Dominance and success was stronger in males. Nonetheless, sex differences in the effect of personality traits in success should be taken with caution, given that our sample only included a small number of females and that one female showed a particularly poor performance.

Our results on Extraversion confirmed our predictions, showing a negative effect of this trait on the probability of success. According to Eysenck’s theory, introverts are more patient and have more goal-oriented attention. Furthermore, introverts’ higher levels of cortical arousal allow them to sustain their attention even under less stimulating conditions (Eysenck, 1981). In our study, puzzle boxes required animals to persist in assembling the different components without getting any reward until they completely solved the task. Therefore, more introverted individuals might have been advantaged when solving these tasks. These results are in line with previous research on humans, showing a negative relationship between Extraversion and academic performance, possibly because extraverts are more social, easily distracted and impulsive (Chamorro-Premuzic & Furnham, 2003; Sánchez, Rejano & Rodríguez, 2001), but also more reward sensitive (Depue & Collins, 1999; Smillie, 2013). However, the effect of Extraversion in non-human primates is, to date, more controversial. Altschul et al. (2017), for instance, found that chimpanzees scoring higher in Extraversion were more accurate in a touchscreen cognitive task. Furthermore, studies on macaques (*Macaca mulatta* and *Macaca fascicularis*) showed a link between success in cognitive tasks and being “active” or “friendly” (Altschul, Terrace & Weiss, 2016; Wergård et al., 2016), which are adjectives that load onto the trait Extraversion. In our study, however, subjects had to be isolated from the social group during the test, and this might have also contributed to the negative association we found between higher Extraversion and success. In particular, more introverted individuals might have been less disturbed by isolation, and might have been more likely to focus on solitary activities. Also, this may be especially true for the chimpanzees in our study, as they are rarely isolated from their group and, with the exception of the experiments described in this study, they hardly ever participate in testing sessions.

In contrast with our predictions, Neuropsychoticism was linked to higher probability of success in females. Nonetheless, as expected, higher scores on this trait slightly increased the probability of success in males. Studies in non-human primates have failed to report any relationship between Neuroticism and cognitive performance (Altschul et al., 2017; Morton, Lee & Buchanan-Smith, 2013). However, in an experiment on social learning in wild baboons (*Papio ursinus*), Carter et al. (2014) reported that more anxious individuals were more likely to improve their performance in a hidden-object task after watching a demonstrator. In contrast, Schubiger et al. (2015) found that male marmosets (*Callithrix jacchus*) showing higher emotional reactivity towards the experimenter (i.e., highly neurotic individuals) were less likely to participate in cognitive tasks, but this did not affect their performance. In humans, higher Neuroticism and higher Psychoticism have been repeatedly linked to poorer performance, both in academic (Chamorro-Premuzic & Furnham, 2003; Flores-Mendoza et al., 2013; Poropat, 2011) and non-academic contexts (Dobson, 2000; Reynolds, McClelland & Furnham, 2014), and especially under stressful conditions (Byrne, Silasi-Mansat & Worthy, 2015). Nonetheless, Eysenck (1981) suggested that the relationship between Neuroticism and performance depends on the intelligence of the subject: higher Neuroticism is related to higher academic achievement in more intelligent individuals, who are better able to cope with anxiety, while the opposite pattern is observed for less intelligent subjects. Other researchers have suggested that neurotics are more creative problem-solvers, because they tend to think about different possibilities and scenarios when they face a new situation (Perkins et al., 2015). Similarly, the dimension Psychoticism in humans includes adjectives such as “imaginative” (Goldberg & Rosolack, 1994), and it has been linked to creativity (Abraham et al., 2005; Acar & Runco, 2012; Eysenck, 1995).

Unsurprisingly, Neuropsychoticism was positively associated with the probability of losing motivation in both sexes, but again this effect was stronger in females. In our study, the puzzle boxes required individuals to be persistent and constant, attributes that are quite opposite to the adjectives that load onto this factor, such as “anxious” and “impulsive” (Úbeda & Llorente, 2015). Therefore, individuals higher in Neuropsychoticism might have been more likely to become anxious and frustrated during the task, ultimately resulting in loss of motivation. Earlier research in chimpanzees has linked higher Neuroticism with the production of self-directed behaviors (a common indicator of anxiety) during cognitive tasks (Herrelko, Vick & Buchanan-Smith, 2012). These findings appear consistent with research in humans, in which Neuroticism has been associated with high levels of tension (Zajenkowska, Zajenkowski & Jankowski, 2015) and test anxiety (Zeidner & Matthews, 2000). On the other hand, Psychoticism in humans is not only related to impulsivity (Chico et al., 2003; Eysenck et al., 1985), but also to low persistence and lack of cooperation (Howarth, 1986). Although the puzzle boxes did not require cooperative behavior, they did require collaboration with the experimenter and the keeper, who were always present during the experimental sessions and interacted with the puzzle boxes in some conditions. Therefore, being more collaborative might have favored motivation in our study. Likewise, this could also explain why, for both sexes, Extraversion was negatively linked to the probability of losing contact with the task. Initially, more extraverted individuals might have been less predisposed to leave their group to participate in the testing sessions. However, once the chimpanzee was in the experimental area, he received the attention of the experimenter, and more importantly, of a familiar keeper. In contrast, Dominance was found to be positively associated with the probability of losing contact with the task, suggesting that dominant chimpanzees might have been less interested in the testing sessions and more eager to return to their group. Finally, our results showed that younger individuals were more likely to lose motivation and stop manipulating the tasks, perhaps because they were more active and attentive to their surroundings, and thus more susceptible to distraction (Riopelle & Rogers, 1965). Studies with larger samples of non-human primates have indeed reported controversial results regarding the effect of age on interest and motivation towards new stimuli (Almeling et al., 2016; Bliss-Moreau & Baxter, 2019; Massen et al., 2013). Our results may be also explained by the characteristics of our study sample, which included 4 juveniles and otherwise relatively young adults (all <28 years), but no older individuals. Therefore, a negative effect of aging described by some authors (Almeling et al., 2016) would have been, by all means, impossible to detect.

Overall, regardless of personality, participation and success were considerably high (above 80% and 90% respectively), and chimpanzees lost contact with the task in only 5% of the trials in which they participated. This suggests that our study subjects were highly interested in the puzzle boxes: they made considerable efforts to solve them, and were often successful. Besides participation, latency was also not related to any personality trait. These results, however, were not entirely unexpected. Firstly, in one of the few studies assessing this measure, Hopper et al. (2014) also failed to report any link between personality traits and latency to success. Moreover, in our study, latency was highly influenced by the fact that a limited time was given to the chimpanzees to solve the tasks, and this time increased with complexity. Thus, as we anticipated, subjects spent more time solving complex than intermediate tasks, and more time solving intermediate than simple tasks. In other words, task complexity was the most important factor predicting latency. In this study, the time given to subjects to solve the puzzle boxes was deemed to be sufficient and in accordance with their level of difficulty. Nonetheless, it is impossible to tell whether, given the opportunity, subjects would continue trying to solve the boxes and if so, for how long.

To our knowledge, this study is the first to link Eysenck’s personality dimensions with cognition in non-human primates, providing some theoretical and practical advantages. Firstly, the PEN model (Eysenck, 1967) associates personality traits with the functioning and structure of cortical and limbic brain regions (Mitchell & Kumari, 2016), which facilitates the understanding of non-human primates personality from an evolutionary and neurobiological perspective. On the other hand, and in contrast with other rating models, the questionnaire we used is less time consuming for the raters, as it includes only 12 adjectives to evaluate. This is particularly useful, considering that most raters are animal keepers who usually lack the time to dedicate to research activities. Therefore, shorter questionnaires can be especially advantageous to evaluate personality in zoos and sanctuaries (Hopper & Cronin, 2018). Nonetheless, we are aware of the limits of the PEN model, which lacks traits like Openness or Conscientiousness (which are described in the FFM), that might importantly affect performance in experimental contexts. In the future, more studies should use different personality questionnaires to better assess the link between personality and cognitive performance. Also, rather than comparing personality ratings with spontaneous behavior, as we did in the present study, assessing behavioral patterns in a testing context could provide a complementary approach to the study of personality (Massen et al., 2013).

We would also like to highlight that the purpose of this study was not to establish a link between personality and a specific cognitive ability (Griffin, Guillette & Healy, 2015), as the tasks here described were not designed for this purpose. Furthermore, comparisons with other species need to be taken with caution, as cognitive tasks are done with different procedures and personality is often assessed with different tools across species (see Morton et al., 2013). Finally, our results warn against generalizing cognitive abilities at the species level, particularly if testing a small sample of subjects, as they could substantially differ in their performance due to personality variation. Moreover, other sources of individual differences may also modulate subjects’ performance, such as past experiences (Bard et al., 2014), rearing conditions (Simpson et al., 2019), affective state (Bethell et al., 2012), and genetic variables (Hopkins et al., 2014). Additionally, future studies may also consider assessing rank when studying primate cognition particularly if tasks are presented in a social context (Wergård et al., 2016). This was unfortunately not possible in the present study, due to changes in group composition and dominance hierarchies that occurred throughout the data collection period. Finally, one of the main limitations of this study was the low statistical power due to the small sample, as well as the fact that males and females were unevenly represented, with males greatly outnumbering females. Therefore, we need to be especially cautious when interpreting sex differences in our models. For example, the fact that personality traits more strongly affected performance in females could depend on the small number of females tested, with inter-individual differences having been magnified.

Last but not least, studying the relationship between personality and measures like interest or motivation can have important implications for animal welfare. Given that individuals with different personality profiles may benefit from different types of cognitive enrichment (Carere & Locurto, 2011), understanding individual differences in personality may be transferred to improving management and quality of life in animals under human control, thus having a positive impact on welfare and conservation (Gartner & Weiss, 2018). In line with this, besides personality, future cognitive research involving captive animals should also consider including welfare indicators that can be monitored during experimental testing. Furthermore, cognitive experiments in a social setting should be considered as an alternative to subjects’ isolation, which would increase validity of findings and improve animal welfare (Cronin, 2017).

## Conclusions

In line with our predictions, chimpanzees’ behavior correlated with some of the personality dimensions described by Eysenck’s PEN model, although construct validity was relatively low. Nonetheless, the PEN model offers some practical advantages compared to other questionnaires, being simpler and less time consuming. Moreover, as predicted, personality traits were related to subjects’ performance in an experimental context. In particular, success was negatively related to Extraversion and Dominance, with these associations being more evident in females. Furthermore, Neuropsychoticism was positively associated with success in females, but not in males. As expected, higher Neuropsychoticism was associated with loss of motivation and therefore higher probability of the chimpanzees stopping manipulating the puzzle boxes, especially in females. Additionally, younger chimpanzees, and those rated lower on Extraversion and higher on Dominance were also more likely to stop interacting with the task. Participation and latency were not related to any personality trait. These findings stress the importance of considering personality when assessing cognitive performance in non-human primates, as the outcomes of a particular test may not necessarily reflect the subject’s ability to perform the task, but rather individual differences in personality.

##  Supplemental Information

10.7717/peerj.9707/supp-1 Figure S1 Cognitive tasks used in this study categorized by complexity levels (simple, intermediate and complex) according to the number of elements that needed to be solved

10.7717/peerj.9707/supp-2 Data S1 Data for performance measures and the personality scores used in the statistical analysis

10.7717/peerj.9707/supp-3 Data S2 Raw data from the behavioural observations

10.7717/peerj.9707/supp-4 Table S1 Behavioral catalogue used to monitor chimpanzees’ behvavior at Fundació Mona from 2006 to 2017 (adapted from Llorente et al., 2015)

10.7717/peerj.9707/supp-5 Table S2 Possible combinations of experimental conditions (task complexity, social information and causal information), number of tasks, and trials performed by each subject in each condition

10.7717/peerj.9707/supp-6 Table S3 Spearman correlations between Eysenck’s personality traits and observed behaviors *N* = 14. Significant results are marked in bold (*p* < 0.05; 95% CI do not overlap 0).

10.7717/peerj.9707/supp-7 Table S4 Individual results in the cognitive tasks

10.7717/peerj.9707/supp-8 Questionnaire S1 English version of the personality questionnaire completed by the raters

10.7717/peerj.9707/supp-9 Supplemental Information 1 Original Spanish version of the personality questionnaire completed by the raters

## Additional Information and Declarations

### Data Availability

The following information was supplied regarding data availability: Raw data for performance measures, personality and behaviour are available in the Supplemental Files.
