# Supplementary material for: Personality, cognition and behavior in chimpanzees: a new approach based on Eysenck’s model
Source: PeerJ. 2020 Aug 17;8:e9707. doi: 10.7717/peerj.9707 (PMC7439959; doi:10.7717/peerj.9707)
Supplement: Figure S1 [file peerj-08-9707-s001.pdf]

SIMPLE TASKS

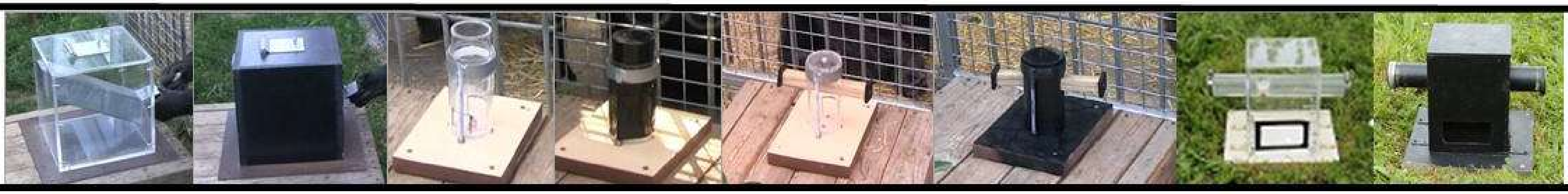

Open Box Moveable Tube Windows Task Tube Cube

INTERMEDIATE TASKS

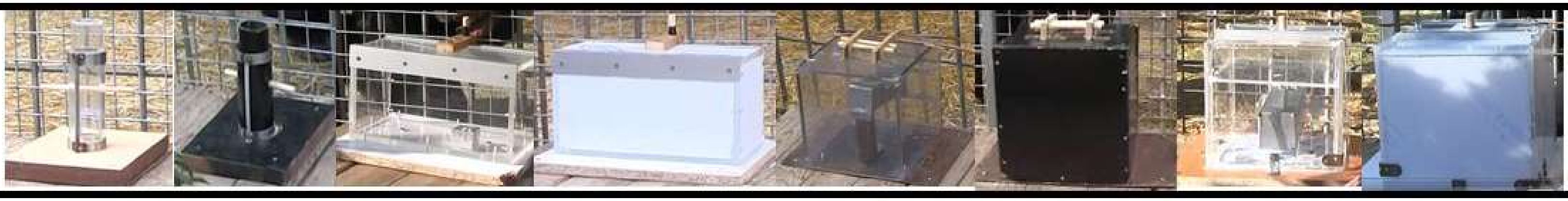

Tower Task Artificial Fruit Food Box Push Box

COMPLEX TASKS

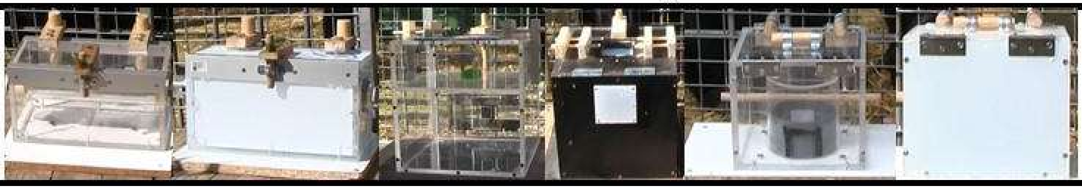

Complex Food Box Complex Fixed Tube Complex Artificial Fruit
