# Supplementary material for: Personality, cognition and behavior in chimpanzees: a new approach based on Eysenck’s model
Source: PeerJ. 2020 Aug 17;8:e9707. doi: 10.7717/peerj.9707 (PMC7439959; doi:10.7717/peerj.9707)
Supplement: Table S1 [file peerj-08-9707-s004.docx]

| **Behavioral category** | **Name of behavior** | **Definition** |
| --- | --- | --- |
| **Solitary** | Abnormal behavior | Maladjusted stereotypical behaviors such as rocking, pacing, self–harm, coprophagy (eating feces), regurgitation, and reingestion, trichotillomania (hair-pulling), trichotillophagia (hair-pulling eating hair), ear-poking, eye-poking. |
|  | Locomotion | Moving from one point to another on a vertical or horizontal surface while not displaying any other behavior in the ethogram. |
|  | Feeding | Searching, locating, handling, and ingesting or transporting food. Includes fluid intake. |
|  | Manipulation | Inspecting elements of the environment or enrichment that are not food with the upper or lower extremities.  Includes transportation and solitary play with objects or enrichment. |
|  | Inactivity | No action or activity, sitting, or lying down. Includes self-observation, yawning, and sleeping. |
|  | Self-directed behavior | Behaviors directed towards the individual, such as self-cleaning, self-grooming, masturbation, scratching, scrubbing, and body inspection. |
|  | Other solitary | Individual behaviors that are not better defined by any other solitary behaviors (e.g. excretion). |
| **Social** | Grooming | Body-cleansing behavior from one individual to another (includes mutual grooming), performed with the upper extremities or with the mouth. |
|  | Agonistic dominance | Threat-related behaviors such as direct aggression, charging display, displacement and resource appropriation (e.g. steal food or objects). Can be accompanied by vocalizations. |
|  | Agonistic submission | Avoiding, food submission (e.g. leave/drop food and move away when others try to steal it), hand-to-mouth, finger-to-mouth. Can be accompanied by vocalizations such as pant-grunts. Includes running away from others in conflict situations. |
|  | Other agonistic | Other behaviors identified as agonistic, but do not fit the criteria of Agonistic dominance or Agonistic submission (e.g. appeasing, consolation, reconciliation, and requesting support). |
|  | Social play | Playful behavior between two or more individuals associated with behavioral indicators of play (e.g. play-face, laugh, friendly head bobbing, soft knocking on the ground, and playful chasing). |
|  | Sexual behavior | Sexual interaction, or search for sexual interaction, between two individuals including behaviors such as: copulation, attempted copulation, genital presentation, and other behaviors directed towards the genitals of another individual. |
|  | Other affiliative | Other behaviors identified as affiliative, but do not fit the criteria of Grooming, Social play or Sexual behavior (e.g. embrace, greetings). |
|  |  |  |
|  | Social proximity | The chimpanzee is at less than one-arm length from one or more subjects, but there is no social interaction between them. |
| **Other** | Not Visible | The chimpanzee or the behavior cannot be identified. |
|  | Not Present | The chimpanzee is not in the outdoor enclosure (e.g. he is in the sleeping areas or in the outdoor cages). |
|  | Human Positive | Affiliative or neutral interaction between chimpanzees and humans (without physical contact). Includes staring at humans from a close distance (1.5m meters from the fence), following humans around the enclosure. |
|  | Human Negative | Agonistic-type interaction or looking for agonistic-type interaction, with humans. |

Llorente M, Riba D, Ballesta S, Feliu O, and Rostán C. 2015. Rehabilitation and socialization of chimpanzees (*Pan troglodytes*) used for entertainment and as pets: An 8-year study at Fundació Mona. *International Journal of Primatology* 36:605-624. DOI: 10.1007/s10764-015-9842-4.
