# Supplementary material for: Personality, cognition and behavior in chimpanzees: a new approach based on Eysenck’s model
Source: PeerJ. 2020 Aug 17;8:e9707. doi: 10.7717/peerj.9707 (PMC7439959; doi:10.7717/peerj.9707)
Supplement: Table S2 [file peerj-08-9707-s005.docx]

| **Task complexity** | **Social information** | **Tasks per**  **subject** | **Causal information (apparatus version)** | **Trials per subject** |
| --- | --- | --- | --- | --- |
| SIMPLE | Control | 1 | Transparent | 4 |
|  |  |  | Opaque | 4 |
|  | No social information | 1 | Transparent | 4 |
|  |  |  | Opaque | 4 |
|  | Social information | 1 | Transparent | 4 |
|  |  |  | Opaque | 4 |
| **TOTAL**  **SIMPLE** |  | **3** |  | **24** |
| INTERMEDIATE | Control | 1 | Transparent | 4 |
|  |  |  | Opaque | 4 |
|  | No social information | 1 | Transparent | 4 |
|  |  |  | Opaque | 4 |
|  | Social information | 1 | Transparent | 4 |
|  |  |  | Opaque | 4 |
| **TOTAL INTERMEDIATE** |  | **3** |  | **24** |
| COMPLEX | Control | 1 | Transparent | 4 |
|  |  |  | Opaque | 4 |
|  | No social information | 1 | Transparent | 4 |
|  |  |  | Opaque | 4 |
|  | Social information | 1 | Transparent | 4 |
|  |  |  | Opaque | 4 |
| **TOTAL**  **COMPLEX** |  | **3** |  | **24** |
