# Supplementary material for: Personality, cognition and behavior in chimpanzees: a new approach based on Eysenck’s model
Source: PeerJ. 2020 Aug 17;8:e9707. doi: 10.7717/peerj.9707 (PMC7439959; doi:10.7717/peerj.9707)
Supplement: Table S3 — N = 14. Significant results are marked in bold (p < 0.05; 95% CI do not overlap 0). [file peerj-08-9707-s006.docx]

|  |  | **Abnormal behavior** | **Locomotion** | **Foraging** | **Manipulation** | **Inactivity** | **Self-directed behavior** | **Other solitary** | **Grooming** | **Agonistic dominance** | **Agonistic submission** | **Other agonistic** | **Social**  **play** | **Sexual behavior** | **Other affiliative** | **Social proximity** | **Human positive** | **Human negative** |
| --- | --- | --- | --- | --- | --- | --- | --- | --- | --- | --- | --- | --- | --- | --- | --- | --- | --- | --- |
| **Extraversion** | **r**  ***p*** | -.275  .342 | .284  .326 | .147  .615 | -.132  .653 | -.433  .122 | -.477  .085 | -.152  .605 | **.705**  **.005** | **.614**  **.020** | -.076  .797 | -.179  .541 | **.692**  **.006** | .449  .108 | 0.310  281 | -.077  .794 | .367  .197 | .499  .069 |
|  | **95% CI** | [-.786, .325] | [-.261, .761] | [-.385, .668] | [-.626,  .429 | [-.881, .375] | [-.837, .163] | [-.649, .436] | **[.147, .957]** | **[.079,**  **.929]** | [-.539, .425] | [-.730, .510] | **[.250,**  **.928]** | [-.053, .830] | [-.268, .796] | [-.607, .468] | [-.182, .732] | [-.086, .900] |
| **Neuropsychoticism** | **r**  ***p*** | .007  .982 | -.336  .240 | **-.640**  **.014** | -.271  .349 | -.015  .958 | .020  .946 | .152  .605 | .099  .737 | 0.211  0.469 | 0.327  0.254 | 0.400  0.157 | -.115  .697 | .449  .108 | 0.143  0.626 | 0.508  0.064 | -.165  .573 | .232  .425 |
|  | **95% CI** | [-.555, .654] | [-.675, .196] | **[-.945, -.172]** | [-.802,  .521] | [-.549, .538] | [-.573, .626] | [-.501, .705] | [-.408, .557] | [-.323, .697] | [-.332, .765] | [-.193, .814] | [-.653, .596] | [-.123, .845] | [-.493, .787] | [-.181, .919] | [-.625,  .381] | [-.303, .711] |
| **Dominance** | **r**  ***p*** | -.442  .114 | .218  .455 | -.055  .852 | -.469  .091 | -.393  .164 | -.429  .126 | -.011  .970 | .547  .043 | **.557**  **.039** | .205  .483 | -.246  .397 | -.084  .776 | .486  .078 | -.160  .584 | .341  .233 | .042  .887 | .404  .151 |
|  | **95% CI** | [-.770, .234] | [-.344, .729] | [-.563, .476] | [-.887,  .170] | [-.791, .307] | [-.833.145] | [-.527, .447] | [-.014, .900] | **[.028,**  **.892]** | [-.366, .688] | [-.693, .334] | [-.623, .454] | [-.096, .873] | [-.642, .460] | [-.389, .791] | [-.674,  .647] | [-.144, .831] |

N=14. Significant results are marked in bold (p<0.05; 95% CI do not overlap 0).
