# Supplementary material for: Personality, cognition and behavior in chimpanzees: a new approach based on Eysenck’s model
Source: PeerJ. 2020 Aug 17;8:e9707. doi: 10.7717/peerj.9707 (PMC7439959; doi:10.7717/peerj.9707)
Supplement: Table S4 [file peerj-08-9707-s007.docx]

4A. Overall results (all tasks). For each subject, sex, age (in 2009), mean and standard deviation ( **±** SD) of participation (0=subject refused to participate, 1=the subject engaged in the task), success (0=failed attempt, 1=successful attempt), latency (in seconds), and percentage of trials in which the subject lost contact with the task.

| **Subject** | **Sex** | **Age** | **Participation** | **Success** | **Latency** | **Lose contact with task** |
| --- | --- | --- | --- | --- | --- | --- |
| África | Female | 10 | 0.94± 0.23 | 0.97± 0.17 | 43.06 ± 67.61 | 0.00 |
| Bea | Female | 24 | 0.83 ± 0.38 | 0.57 ± 0.50 | 13.15 ± 12.84 | 29.17 |
| Bongo | Male | 9 | 0.94± 0.23 | 0.85± 0.36 | 26.70 ± 42.08 | 12.50 |
| Charly | Male | 20 | 0.97 ± 0.17 | 1.00± 0.00 | 47.54 ± 61.63 | 0.00 |
| Coco | Female | 15 | 0.97± 0.17 | 0.99± 0.12 | 29.07 ± 40.97 | 0.00 |
| Juanito | Male | 6 | 1.00± 0.00 | 1.00± 0.00 | 46.46 ± 49.38 | 0.00 |
| Marco | Male | 25 | 0.44± 0.50 | 1.00± 0.00 | 10.56 ± 9.23 | 0.00 |
| Nico | Male | 8 | 0.56± 0.50 | 0.98± 016 | 11.28 ± 15.24 | 0.00 |
| Tico | Mal | 22 | 0.35± 0.48 | 0.76 ± 0.44 | 6.68 ± 4.18 | 8.33 |
| Tom | Male | 24 | 0.78± 0.42 | 0.86 ± 0.35 | 40.02 ± 46.12 | 9.38 |
| Toni | Male | 26 | 1.00± 0.00 | 0.92± 0.28 | 48.58 ± 68.53 | 2.78 |
| Victor | Male | 27 | 0.89± 0.32 | 0.97 ± 0.18 | 31.80 ± 48.07 | 1.39 |
| Waty | Female | 12 | 0.88 ± 0.33 | 0.90 ± 0.30 | 42.42 ± 61.31 | 4.17 |
| ** ± SD** |  |  | **0.81 ± 0.22** | **0.91± 0.13** | **30.55± 15.57** | **5.21± 8.36** |

4B. Results for simple tasks. For each subject, sex, age (in 2009), mean and standard deviation ( ± SD) of participation (0=subject refused to participate, 1=the subject engaged in the task), success (0=failed attempt, 1=successful attempt), latency (in seconds), and percentage of trials in which the subject lost contact with the task.

| **Subject** | **Sex** | **Age** | **Participation** | **Success** | **Latency** | **Lose contact with task** |
| --- | --- | --- | --- | --- | --- | --- |
| África | Female | 10 | 1.00± 0.00 | 0.92± 0.28 | 7.45± 14.47 | 0.00 |
| Bea | Female | 24 | 0.67± 0.48 | 0.88± 0.34 | 7.14± 6.25 | 4.17 |
| Bongo | Male | 9 | 1.00± 0.00 | 0.96± 0.20 | 5.70± 5.41 | 0.00 |
| Charly | Male | 20 | 1.00± 0.00 | 1.00± 0.00 | 4.13± 2.66 | 0.00 |
| Coco | Female | 15 | 0.92± 0.28 | 1.00± 0.00 | 4.64± 3.20 | 0.00 |
| Juanito | Male | 6 | 1.00± 0.00 | 1.00± 0.00 | 15.08± 18.29 | 0.00 |
| Marco | Male | 25 | 1.00± 0.00 | 1.00± 0.00 | 7.13± 3.35 | 0.00 |
| Nico | Male | 8 | 1.00± 0.00 | 0.96± 0.20 | 8.09± 6.91 | 0.00 |
| Tico | Mal | 22 | 0.50± 0.51 | 1.00± 0.00 | 4.92± 2.15 | 0.00 |
| Tom | Male | 24 | 0.83± 0.38 | 0.90± 0.31 | 15.24± 26.70 | 4.17 |
| Toni | Male | 26 | 1.00± 0.00 | 0.92± 0.28 | 8.82± 8.06 | 4.17 |
| Victor | Male | 27 | 1.00± 0.00 | 1.00± 0.00 | 6.58± 6.95 | 0.00 |
| Waty | Female | 12 | 0.67± 0.48 | 0.75± 0.45 | 4.58± 4.44 | 4.17 |
| ** ± SD** |  |  | **0.89± 0.17** | **0.94± 0.07** | **7.65± 3.63** | **1.28± 2.00** |

4C. Results for intermediate tasks. For each subject, sex, age (in 2009), mean and standard deviation ( ± SD) of participation (0=subject refused to participate, 1=the subject engaged in the task), success (0=failed attempt, 1=successful attempt), latency (in seconds) and percentage of trials in which the subject lost contact with the task.

| **Subject** | **Sex** | **Age** | **Participation** | **Success** | **Latency** | **Lose contact with task** |
| --- | --- | --- | --- | --- | --- | --- |
| África | Female | 10 | 1.00± 0.00 | 1.00 ± 0.00 | 15.54 ± 16.95 | 0.00 |
| Bea | Female | 24 | 1.00± 0.00 | 0.83± 0.38 | 17.35 ± 14.64 | 16.67 |
| Bongo | Male | 9 | 1.00± 0.00 | 0.92± 0.28 | 15.18 ± 14.57 | 8.33 |
| Charly | Male | 20 | 1.00± 0.00 | 1.00± 0.00 | 21.17 ± 15.04 | 0.00 |
| Coco | Female | 15 | 1.00± 0.00 | 1.00± 0.00 | 15.63 ± 12.35 | 0.00 |
| Juanito | Male | 6 | 1.00± 0.00 | 1.00± 0.00 | 49.46 ± 56.62 | 0.00 |
| Marco | Male | 25 | 0.33 ± 0.48 | 1.00± 0.00 | 20.88 ± 13.36 | 0.00 |
| Nico | Male | 8 | 0.67 ± 0.48 | 1.00± 0.00 | 15.88 ± 21.92 | 0.00 |
| Tico | Mal | 22 | 0.33 ± 0.00 | 0.88± 0.35 | 9.71 ± 5.19 | 4.17 |
| Tom | Male | 24 | 0.67 ± 0.48 | 0.69± 0.48 | 22.09 ± 42.80 | 20.83 |
| Toni | Male | 26 | 1.00 ± 0.00 | 1.00± 0.00 | 18.79 ± 15.40 | 0.00 |
| Victor | Male | 27 | 0.67 ± 0.48 | 1.00± 0.00 | 10.25 ± 5.14 | 0.00 |
| Waty | Female | 12 | 1.00 ± 0.00 | 0.92± 0.28 | 22.50 ± 42.21 | 8.33 |
| ** ± SD** |  |  | **0.82± 0.26** | **0.94± 0.10** | **19.57 ± 9.86** | **4.49 ± 7.11** |

4D. Results for complex tasks. For each subject, sex, age (in 2009), mean and standard deviation ( ± SD) of participation (0=subject refused to participate, 1=the subject engaged in the task), success (0=failed attempt, 1=successful attempt), latency (in seconds) and percentage of trials in which the subject lost contact with the task.

| **Subject** | **Sex** | **Age** | **Participation** | **Success** | **Latency** | **Lose contact with task** |
| --- | --- | --- | --- | --- | --- | --- |
| África | Female | 10 | 0.83± 0.38 | 1.00± 0.00 | 115.25± 84.56 | 0.00 |
| Bea | Female | 24 | 0.83± 0.38 | 0.00± 0.00 | - | 66.67 |
| Bongo | Male | 9 | 0.83± 0.38 | 0.65± 0.49 | 83.23± 58.52 | 29.17 |
| Charly | Male | 20 | 0.92± 0.28 | 1.00± 0.00 | 123.00± 57.53 | 0.00 |
| Coco | Female | 15 | 1.00± 0.00 | 0.96± 0.20 | 66.48± 52.55 | 0.00 |
| Juanito | Male | 6 | 1.00± 0.00 | 1.00± 0.00 | 91.87± 32.68 | 0.00 |
| Marco | Male | 25 | 0.00± 0.00 | - | - | 0.00 |
| Nico | Male | 8 | 0.00± 0.00 | - | - | 0.00 |
| Tico | Mal | 22 | 0.21± 0.41 | 0.00± 0.00 | - | 20.83 |
| Tom | Male | 24 | 0.88± 0.34 | 1.00± 0.00 | 84.21± 35.24 | 0.00 |
| Toni | Male | 26 | 1.00± 0.00 | 0.83± 0.38 | 128.05± 78.17 | 4.17 |
| Victor | Male | 27 | 1.00± 0.00 | 0.92± 0.28 | 75.00± 60.03 | 4.17 |
| Waty | Female | 12 | 0.96± 0.20 | 1.00± 0.00 | 81.22± 71.45 | 0.00 |
| ** ± SD** |  |  | **0.73± 0.38** | **0.76± 0.39** | **94.26± 22.22** | **9.62 ± 19.50** |
