# Supplementary material for: Personality, cognition and behavior in chimpanzees: a new approach based on Eysenck’s model
Source: PeerJ. 2020 Aug 17;8:e9707. doi: 10.7717/peerj.9707 (PMC7439959; doi:10.7717/peerj.9707)
Supplement: Questionnaire S1 [file peerj-08-9707-s008.docx]

**PERSONALITY QUESTIONNAIRE FOR CHIMPANZEES**

**INSTRUCTIONS:**

- Please answer the questionnaire individually, without discussing it with other keepers, volunteers or researchers.

- Evaluate each personality trait by assigning a numerical value on a scale of 1 to 7, depending on whether the subject is better identified with one end of the scale or the other. Example for a subject considered “social”:

Antisocial 1 2 3 4 5 6 7 Social

1: Very antisocial 2: Antisocial 3: Slightly antisocial 4: Neutral 5: Slightly social 6: Social 7: Very social

- It is important to evaluate all the adjectives for all the subjects. Some traits might be difficult to assess, and some may be very similar. However, in order to obtain a complete personality profile of each individual, it is highly desirable that you evaluate all adjectives. Nevertheless, if you experience a great difficulty assessing a certain adjective, you can leave it blank.

-Each item should be evaluated by focusing on the two opposite adjectives. If necessary, at the end of the document you will find explanatory definitions for all the adjectives used (with their antonyms in brackets), in order to facilitate the assessment.

- Traits involving social aspects can refer to how subjects behave with humans or with their conspecifics (other chimpanzees), and assessment of these traits should include both. Nevertheless, if the subject behaves differently with humans than with other chimpanzees, the evaluation should focus on its relationships with conspecifics.

**NAME OF THE CHIMPANZEE:**

| Social | 1 2 3 4 5 6 7 | Antisocial |
| --- | --- | --- |
| Active | 1 2 3 4 5 6 7 | Passive |
| Dominant | 1 2 3 4 5 6 7 | Submissive |
| Spontaneous | 1 2 3 4 5 6 7 | Not spontaneous |
| Calm | 1 2 3 4 5 6 7 | Anxious |
| Good-tempered | 1 2 3 4 5 6 7 | Bad-tempered |
| Brave | 1 2 3 4 5 6 7 | Fearful |
| Cheerful | 1 2 3 4 5 6 7 | Sad |
| Pacific | 1 2 3 4 5 6 7 | Aggressive |
| Cautious | 1 2 3 4 5 6 7 | Impulsive |
| Empathic | 1 2 3 4 5 6 7 | Cruel |
| Not creative | 1 2 3 4 5 6 7 | Creative |

**ADJECTIVES’ DEFINITIONS:**

**Social:** The subject enjoys interacting with others (≠Antisocial)

**Active:** Energetic, engaging in physical activity (≠Passive)

**Dominant:** The subject exercises power over the other individuals (≠Submissive)

**Spontaneous:** The subject has an open, natural and uninhibited behaviour (≠Not spontaneous)

**Calm:** The subject does not show tension or anger (≠Anxious)

**Good-tempered:** Naturally friendly and pleasant (≠Bad-tempered)

**Brave:** Determined, daring (≠Fearful)

**Cheerful:** The subject expresses joy and happiness (≠Sad)

**Pacific:** The subject does not start conflicts, nor participates in them (≠Aggressive)

**Cautious:** Forehanded, thoughtful (≠Impulsive)

**Empathic:** The subject shows concern for the states and needs of others (≠Cruel)

**Creative:** The subject is able to produce new ideas, solve and find answers to new problems (≠Not creative).
