# Supplementary material for: Personality, cognition and behavior in chimpanzees: a new approach based on Eysenck’s model
Source: PeerJ. 2020 Aug 17;8:e9707. doi: 10.7717/peerj.9707 (PMC7439959; doi:10.7717/peerj.9707)
Supplement: Supplemental Information 1 [file peerj-08-9707-s009.docx]

**CUESTIONARIO PARA EVALUAR LA PERSONALIDAD EN PRIMATES NO HUMANOS**

**INSTRUCCIONES:**

- Responda al cuestionario de manera individual, sin comentarlo con sus compañeros.

- Evalúe los rasgos de personalidad marcando el valor que crea más adecuado en la puntuación de la escala de intensidad, dependiendo de si identifica más a un individuo en un extremo del eje o en el otro. Ejemplo para un individuo social:

Antisocial 1 2 3 4 5 6 7 Social

1: Muy antisocial 2: Antisocial 3: Poco antisocial 4: Neutro 5: Poco social 6: Social 7: Muy social

- Es importante evaluar todos los adjetivos para todos los sujetos de estudio. Entendemos que ciertos adjetivos pueden ser difíciles de valorar, además de muy parecidos. Sin embargo, es muy importante para obtener un buen perfil de personalidad de los sujetos intentar evaluar todos los adjetivos. Aun así, si encuentra demasiada dificultad para evaluar algún adjetivo, puede dejar el espacio en blanco.

- Los cuestionarios están diseñados para poder ser evaluados únicamente con los adjetivos bipolares propuestos. No obstante, en caso necesario, al final de este documento podrá encontrar unas definiciones aclaratorias de los adjetivos utilizados (con sus respectivos antónimos entre paréntesis) para que le faciliten la evaluación.

- Ciertos adjetivos de carácter social pueden hacer referencia a cómo se comportan los sujetos en su relación con los humanos o en relación con los conespecíficos (otros chimpancés). La evaluación de estos adjetivos de carácter social deberá incluir ambos ámbitos. No obstante, ante determinados contextos un sujeto puede comportarse de manera opuesta (Ej. un sujeto se muestra de manera social con humanos y en un modo asocial con conespecíficos), en dichas ocasiones aunque se persiga una valoración global, prevalecerá su relación con sus congéneres, frente a su relación con los humanos.

**NOMBRE DEL CHIMPANCÉ:**

| Social *(Social)* | 1 2 3 4 5 6 7 | Antisocial *(Unsocial)* |
| --- | --- | --- |
| Activo *(Active)* | 1 2 3 4 5 6 7 | Pasivo *(Passive)* |
| Dominante *(Dominant)* | 1 2 3 4 5 6 7 | Sumiso *(Submissive)* |
| Espontaneo *(Spontaneous)* | 1 2 3 4 5 6 7 | No espontaneo *(Not spontaneous)* |
| Tranquilo *(Calm)* | 1 2 3 4 5 6 7 | Ansioso *(Anxious)* |
| Con buen humor *(Good-tempered)* | 1 2 3 4 5 6 7 | Mal humorado *(Bad-tempered)* |
| Valiente (Brave) | 1 2 3 4 5 6 7 | Temeroso *(Fearful)* |
| Alegre *(Happy, cheerful)* | 1 2 3 4 5 6 7 | Triste *(Sad)* |
| Pacífico *(Not aggressive)* | 1 2 3 4 5 6 7 | Agresivo *(Aggressive)* |
| Cauto *(Not impulsive)* | 1 2 3 4 5 6 7 | Impulsivo *(Impulsive)* |
| Empático *(Empathic)* | 1 2 3 4 5 6 7 | Cruel *(Cruel)* |
| No creativo *(Not creative)* | 1 2 3 4 5 6 7 | Creativo *(Creative)* |

**DEFINICIONES ADJETIVOS:**

**Social:** Le gusta relacionarse con otros (≠Antisocial)

**Activo:** Enérgico, que actúa con energía o desarrolla gran actividad (≠Pasivo)

**Dominante:** Que ejerce poder sobre el resto de individuos (≠Sumiso)

**Espontáneo**: Que manifiesta comportamientos voluntarios, de forma natural (≠No espontáneo)

**Tranquilo:** Sosegado, calmado (≠Ansioso)

**Con buen humor**: Animado, jovial (≠Malhumorado)

**Valiente:** Decidido, atrevido (≠Temeroso)

**Alegre:** Que manifiesta o expresa alegría, animado, contento (≠Triste)

**Pacífico:** Que no participa ni crea conflicto (≠Agresivo)

**Cauto:** Cauteloso, precavido (≠Impulsivo)

**Empático:** Que muestra preocupación por el estado y las necesidades de los demás (≠Cruel)

**Creativo:** Capaz de producir idas nuevas, solucionar y buscar respuesta a nuevos problemas (≠No creativo)
